# Direct specification of lymphatic endothelium from mesenchymal progenitors

**DOI:** 10.1038/s44161-024-00570-5

**Published:** 2025-01-02

**Authors:** Irina-Elena Lupu, David E. Grainger, Nils Kirschnick, Sarah Weischer, Erica Zhao, Ines Martinez-Corral, Hans Schoofs, Marie Vanhollebeke, Grace Jones, Jonathan Godwin, Aden Forrow, Ines Lahmann, Paul R. Riley, Thomas Zobel, Kari Alitalo, Taija Mäkinen, Friedemann Kiefer, Oliver A. Stone

**Affiliations:** 1 Department of Physiology, Anatomy & Genetics, University of Oxford, Oxford, UK; 2 Institute of Developmental & Regenerative Medicine, University of Oxford, Oxford, UK; 3 University of Münster, European Institute for Molecular Imaging, Münster, Germany; 4 Max Planck Institute for Molecular Biomedicine, Münster, Germany; 5 University of Münster, Cells in Motion Interfaculty Centre, Imaging Network, Münster, Germany; 6 Department of Immunology, Genetics and Pathology, Uppsala University, Uppsala, Sweden; 7 Mathematical Institute, University of Oxford, Oxford, UK; 8 Developmental Biology/Signal Transduction, Max-Delbrück-Center for Molecular Medicine, Berlin, Germany; 9 Neurowissenschaftliches Forschungzentrum, NeuroCure Cluster of Excellence, Charité-Universitätsmedizin Berlin, Berlin, Germany; 10 Wihuri Research Institute, Helsinki, Finland; 11 Translational Cancer Medicine Program, Biomedicum Helsinki, University of Helsinki, Helsinki, Finland

**Keywords:** Lymphangiogenesis, Stem-cell differentiation, Differentiation, Cell lineage, Cell lineage

## Abstract

During embryogenesis, endothelial cells (ECs) are generally described to arise from a common pool of progenitors termed angioblasts, which diversify through iterative steps of differentiation to form functionally distinct subtypes of ECs. A key example is the formation of lymphatic ECs (LECs), which are thought to arise largely through transdifferentiation from venous endothelium. Opposing this model, here we show that the initial expansion of mammalian LECs is primarily driven by the in situ differentiation of mesenchymal progenitors and does not require transition through an intermediate venous state. Single-cell genomics and lineage-tracing experiments revealed a population of paraxial mesoderm-derived *Etv2*^*+*^*Prox1*^*+*^ progenitors that directly give rise to LECs. Morphometric analyses of early LEC proliferation and migration, and mutants that disrupt lymphatic development supported these findings. Collectively, this work establishes a cellular blueprint for LEC specification and indicates that discrete pools of mesenchymal progenitors can give rise to specialized subtypes of ECs.

## Abstract (web summary)

Lupu, Grainger, Kirschnick et al. show that, contrary to prevailing belief, the initial specification of mammalian lymphatic endothelial cells primarily occurs from previously unidentified mesenchymal angioblasts rather than from venous endothelium.

## Main

The lymphatic vasculature is a blunt-ended, unidirectional vessel network essential for the maintenance of tissue fluid homeostasis, immune cell trafficking and lipid absorption^1^. Although defects in the formation and function of lymphatic vessels have been described in a broad range of diseases, including lymphedema, cancer, obesity and neurodegeneration^1^, our understanding of the mechanisms governing their formation and function has historically trailed behind that of the cardiovascular system^2^. The inner lining of lymphatic vessels is formed by a single layer of lymphatic endothelial cells (LECs), which have been shown to play important roles in organ development^3^, homeostasis^4^ and regeneration^3^. Despite these key functions, our understanding of how LECs are specified during embryonic development remains incomplete, with the developmental origins of LECs debated for over a century^5–11^.

During embryogenesis, formation of the vertebrate vasculature begins with the de novo specification of endothelial cells (ECs) from mesoderm-derived mesenchymal progenitors known as angioblasts^12,13^. In response to locally deposited growth factors, angioblasts further differentiate to form ECs that migrate and coalesce to form the first functional vessels. It is from these initial vessels that most blood and lymphatic vessel networks are thought to emerge^14^. Early histological assessment of lymphatic vessel formation in mammalian embryos described venous endothelium^8^ and mesenchymal cells^5^ as sources of lymphatics. However, loss-of-function^15^ and genetic lineage-tracing^9^ analyses in mouse later indicated that LECs mainly transdifferentiate from venous endothelium, with live imaging of lymphatic development in zebrafish showing that a similar process occurs in teleosts^7,11^. In contrast, live imaging of facial lymphatic specification in zebrafish^16^, chimeric transplantation analyses in avian embryos^10^ and distinct lineage-tracing analyses in mouse^6,17^ revealed alternative non-venous sources of LECs in various tissues.

Using genetic lineage tracing in mouse, we previously showed that the overarching source of most mammalian LECs is the paraxial mesoderm^18^. These detailed imaging analyses revealed that paraxial mesoderm-derived cells contribute to venous endothelium and form LECs in most tissues. Here, using a combination of single-cell genomics, inducible lineage tracing and high-resolution imaging, we show that LECs can arise directly from a specialized pool of *Etv2*^*+*^*Prox1*^*+*^ angioblasts, in a spatiotemporal pattern that mimics migration from venous endothelium into the surrounding mesenchyme.

## Results

### Single-cell analysis of EC specification

**Fig. 1:**
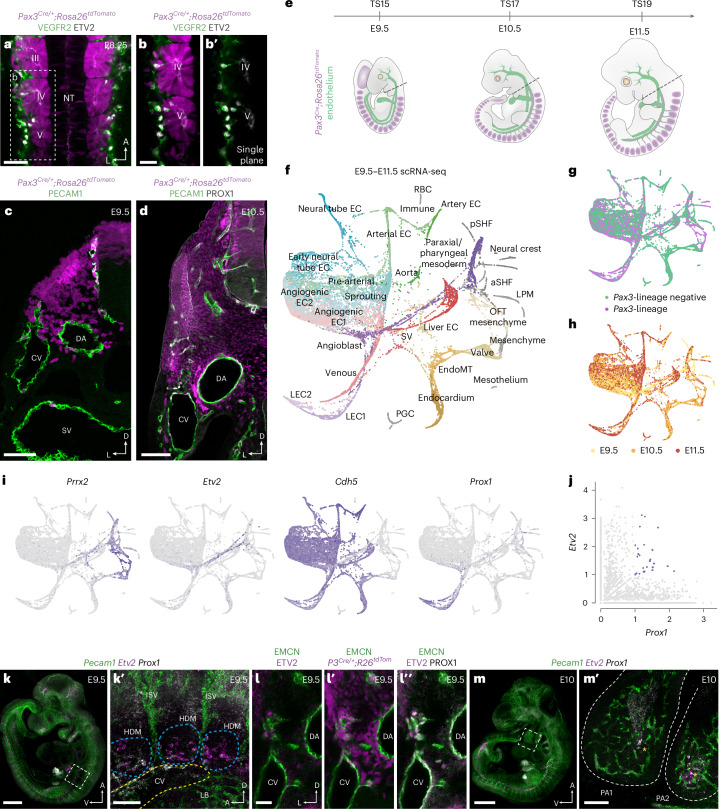
Spatiotemporal analyses of EC specification. **a**, Representative whole-mount immunofluorescence for tdTomato, VEGFR2 and ETV2 in an E8.25 *Pax3*^*Cre/+*^*;Rosa26*^*tdTomato*^ embryo. Dorsal view at the level of somites III–V (*n* = 4). **b**,**b′**, High-magnification single confocal plane of boxed area in **a**, highlighting a population of VEGFR2^+^ETV2^+^ angioblasts emerging from the lineage-traced somite. **c**, Representative immunofluorescence for tdTomato and PECAM1 on a transverse cryosection from a *Pax3*^*Cre/+*^*;Rosa26*^*tdtomato*^ embryo at E9.5 (*n* = 6). **d**, Representative immunofluorescence for tdTomato, PECAM1 and PROX1 on a transverse vibratome section from a *Pax3*^*Cre/+*^*;Rosa26*^*tdTomato*^ embryo at E10.5 (*n* = 6). **e**, Schematic highlighting embryonic stages and dissection strategy (dashed line) for scRNA-seq analyses. **f**, ForceAtlas2 (FA) embedding of 19,699 cells based on PAGA, with each dot representing a single cell. Cellular states were manually annotated based on known gene expression patterns. **g**, FA embedding showing the relationship between cell lineage and cell state. **h**, FA embedding showing the relationship between embryonic stage and cell state. **i**, FA embedding showing gene expression of mesenchymal (*Prrx2*), angioblast (*Etv2*), pan-endothelial (*Cdh5*) and lymphatic endothelial (*Prox1*) markers. **j**, Scatterplot showing co-expression of *Etv2* and *Prox1* in single cells. **k**,**k′**, Representative whole-mount analysis of *Pecam1*, *Etv2* and *Prox1* expression at E9.5 using HCR shows *Etv2* and *Prox1* co-expressing cells in the hypaxial somite (*n* = 6). **l**–**l**″, Representative immunofluorescence for EMCN, ETV2, PROX1 and tdTomato on transverse vibratome sections from a *Pax3*^*Cre/+*^*;Rosa26*^*tdtomato*^ embryo at E9.5 (*n* = 6). **m**,**m′**, Representative whole-mount analysis of *Pecam1*, *Etv2* and *Prox1* expression at E10.0 using HCR shows *Etv2* and *Prox1* co-expressing cells in the pharyngeal arches (*n* = 6). TS, Thieler stage; aSHF, anterior second heart field; CCV, common cardinal vein; DA, dorsal aorta; HDM, hypaxial dermomyotome; ISV, intersegmental vessel; LB, limb bud; LPM, lateral plate mesoderm; NC mesenchyme, neural crest-derived mesenchyme; NT, neural tube; OFT, outflow tract; PA, Pharyngeal arch; PGC, primordial germ cell; pSHF, posterior second heart field; RBC, red blood cell; SHF, second heart field; SV, sinus venosus. Scale bars, 50 μm (**a**, **c** and **l**–**l**″), 25 μm (**b**), 100 μm (**d**, **k′** and **m′**), 500 μm (**k**), 1 mm (**m**).

**Extended Data Fig. 1:**
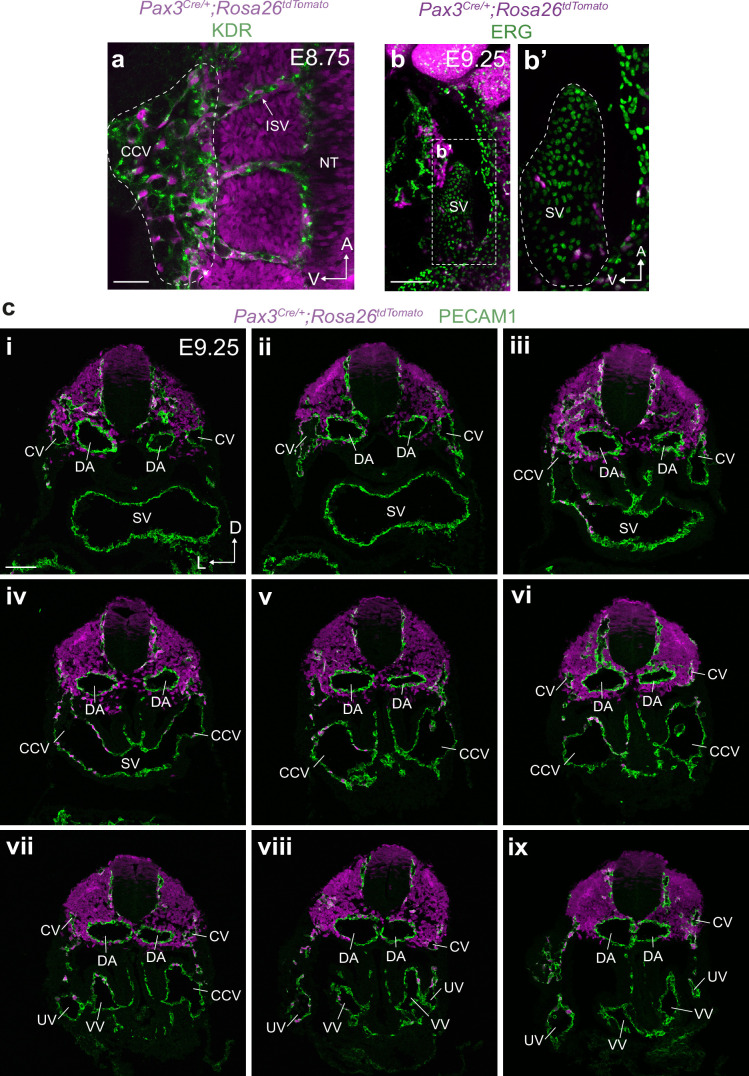
Contribution of *Pax3*-lineage cells to the developing vasculature. (**a**) Representative whole mount immunofluorescence for tdTomato and VEGFR2 in a *Pax3*^*Cre/+*^*;Rosa26*^*tdTomato*^ embryo at E8.75. Lateral view at the level of somites II-V (n = 4). (**b**) Representative whole mount immunofluorescence for ERG and tdTomato at the level of the sinus venosus in a *Pax3*^*Cre/+*^*;Rosa26*^*tdTomato*^ embryo at E9.25 (n = 4). (**b′**) High magnification view of boxed area in **b**. (**c**) Representative immunofluorescence for PECAM1 and tdTomato on transverse cryosections spanning anterior (i) to posterior (ix) regions of an individual *Pax3*^*Cre/+*^*;Rosa26*^*tdTomato*^ embryo at E9.25 (n = 3). (ISV, intersegmental vessel; CCV, common cardinal vein; SV, sinus venosus; CV, cardinal vein; DA, dorsal aorta; UV, umbilical vein; VV, vitelline vein. Scale bars - 50 μm (a,c), 100 μm (b)).

**Extended Data Fig. 2:**
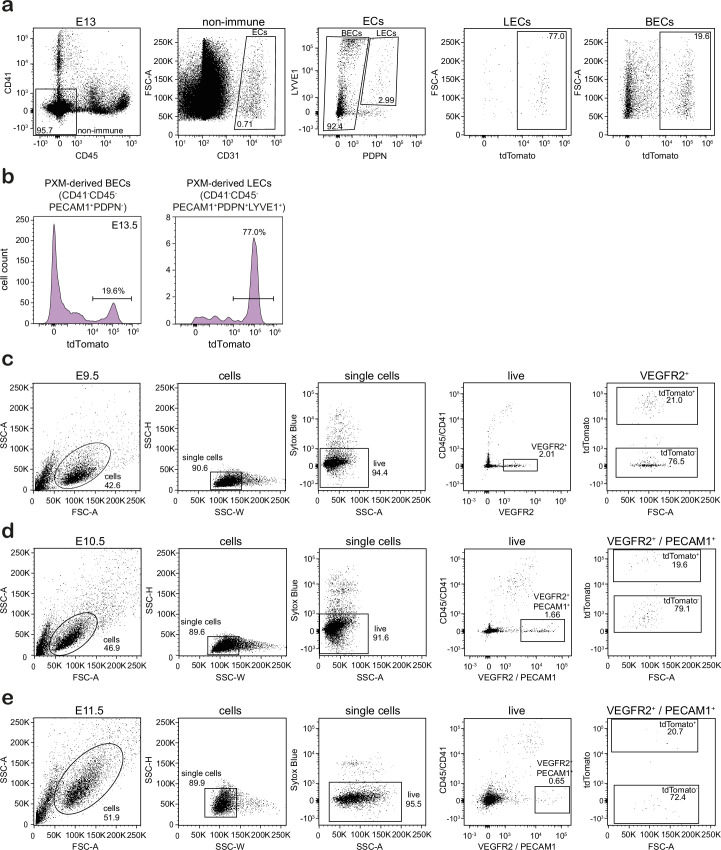
Flow cytometry and FACS sorting strategies. (**a**) Flow cytometry analysis of tdTomato labelling of LECs and BECs in *Pax3*^*Cre/+*^*;Rosa26*^*tdTomato*^ embryos at E13.5. (**b**) Flow cytometry analysis of tdTomato labelling of blood endothelial cell (BEC) and lymphatic endothelial cell (LEC) populations in *Pax3*^*Cre/+*^*;Rosa26*^*tdTomato*^ embryos at E13.5. Gating strategy to sort individual cells from *Pax3*^*Cre/+*^*;Rosa26*^*tdTomato*^ embryos for scRNA-seq at (**c**) E9.5, (**d**) E10.5 and (**e**) E11.5.

Immunofluorescence analyses of *Pax3*^*Cre/+*^*;Rosa26*^*tdTomato*^ embryos, where paraxial mesoderm-derived LECs and their ancestors are labeled by tdTomato^18^, revealed that VEGFR2^+^ETV2^+^ angioblasts begin to emerge from somitic mesoderm at embryonic day (E) 8.25 (Fig. 1a-b′). Subsequently, paraxial mesoderm-derived cells contribute to the common cardinal vein (CV) and intersegmental vessels at E8.75 (Extended Data Fig. 1a), the sinus venosus, cardinal, umbilical and vitelline veins at E9.25 (Extended Data Fig. 1b,c) and E9.5 (Fig. 1c) and PROX1^+^ ECs sitting both inside and outside of the CV at E10.5 (Fig. 1d). Flow cytometry analysis of dissected *Pax3*^*Cre/+*^*;Rosa26*^*tdTomato*^ embryos at E13.5 showed that, although the proportion of paraxial mesoderm-derived blood ECs (BECs) is relatively limited (Extended Data Fig. 2a,b), most LECs are paraxial mesoderm derived at this developmental stage (Extended Data Fig. 2a,b).

**Extended Data Fig. 3:**
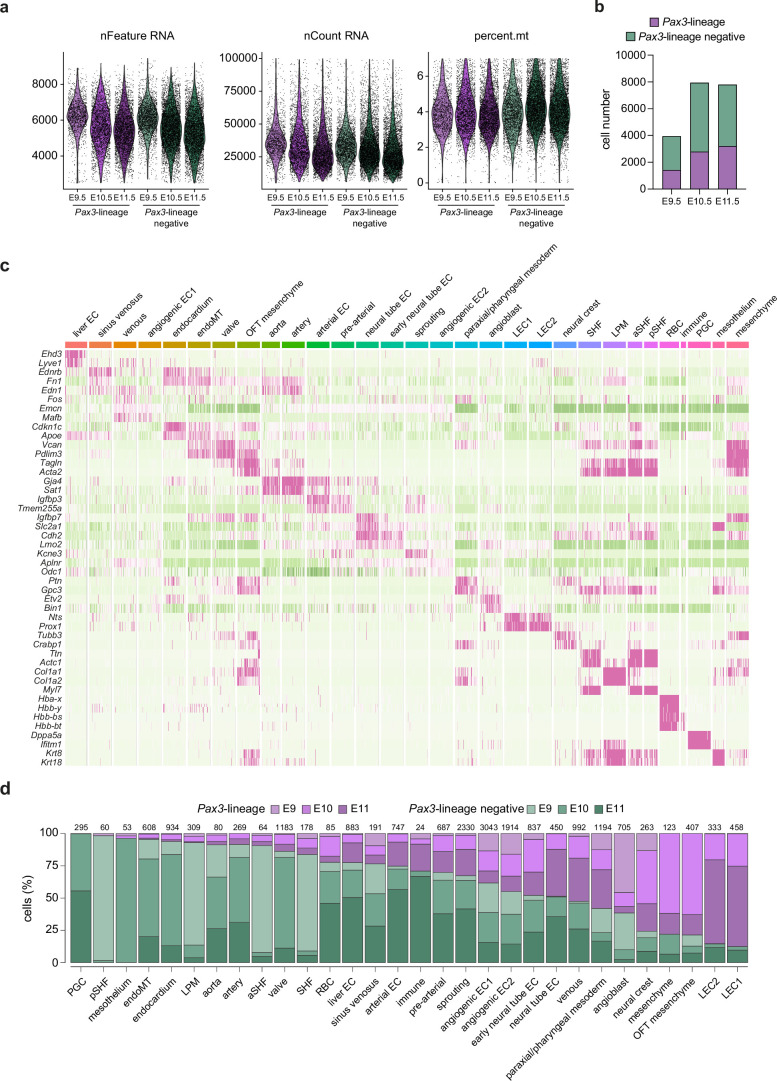
scRNA-seq Quality Control. (**a**) Quality control (QC) plots showing RNA features, counts and percentage of mitochondrial reads per cell. Cells with less than 2500 detected features (genes), more than 100,000 UMIs (counts) and 7% of mitochondrial reads were excluded from downstream analyses. (**b**) Histogram showing the number of single cells from each lineage and stage that passed QC. (**c**) Heatmap showing normalized expression of two diagnostic markers for each cell state. (**d**) Histogram showing the number and percentage of single cells from each lineage and stage assigned to each cell state. (EC, endothelial cell; OFT, outflow tract; LEC, lymphatic endothelial cell; SHF, second heart field; LPM, lateral plate mesoderm; NC mesenchyme, neural crest derived mesenchyme; aSHF, anterior second heart field; pSHF, posterior second heart field; RBC, red blood cell; PGC, primordial germ cell).

**Extended Data Fig. 4:**
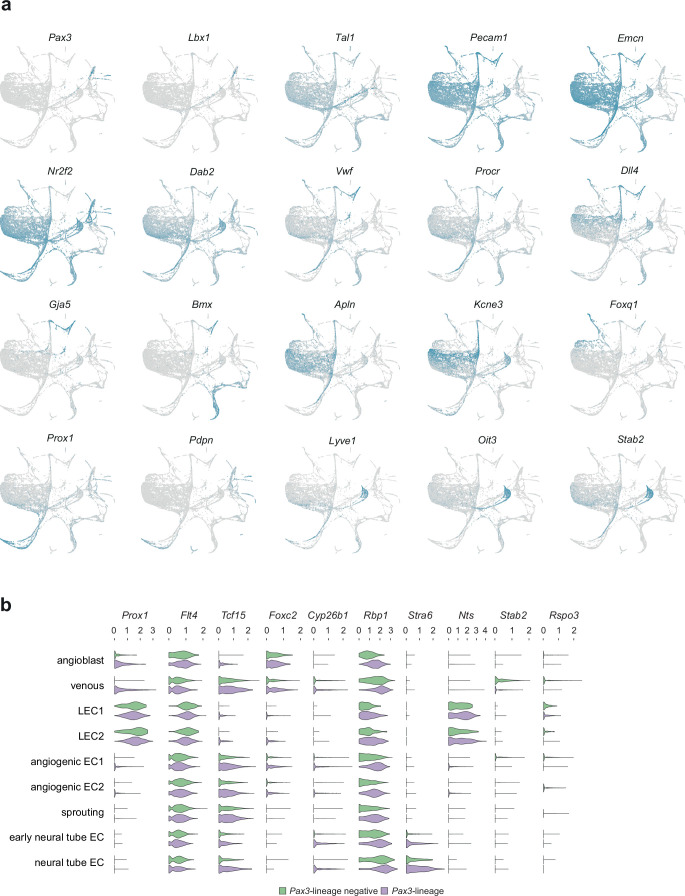
scRNA-seq gene expression analyses. (**a**) ForceAtlas2 (FA) embedding showing expression of somitic paraxial mesoderm (*Pax3*, *Lbx1*), angioblast/early EC (*Tal1*), endothelial (*Pecam1, Emcn*), venous (*Nr2f2, Dab2*), large vessel (*Vwf*, *Procr*), arterial (*Dll4*, *Gja5, Bmx*), angiogenic EC (*Apln*, *Kcne3*), neural tube EC (*Foxq1*), lymphatic EC (*Prox1*, *Pdpn, Lyve1*) and sinus venosus/liver EC (*Oit3*, *Stab2*) markers. (**b**) Violin plots showing expression of selected genes in *Pax3*-lineage negative and *Pax3*-lineage positive cells across indicated cellular states.

**Extended Data Fig. 5:**
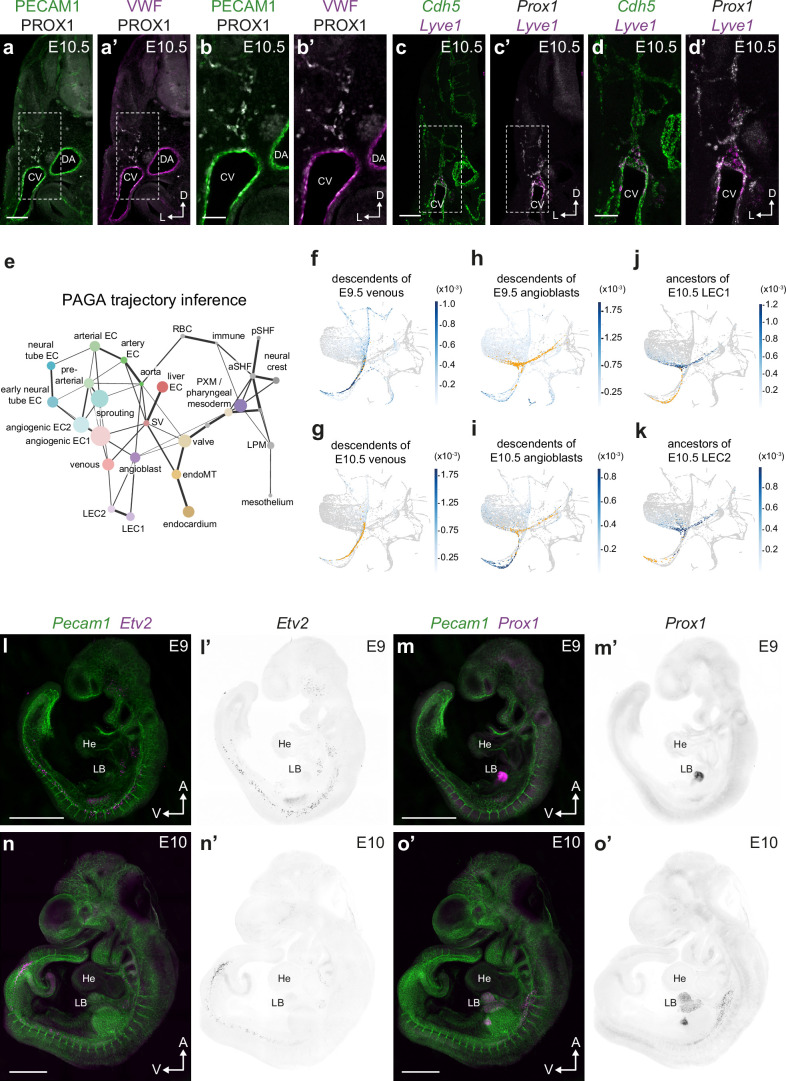
Gene expression and trajectory analyses. Representative immunofluorescence images of PECAM1 and PROX1 (**a**,**b**) or VWF and PROX1 (**a′**,**b′**) on a transverse vibratome section from an E10.5 embryo (**b**-**b′** - high magnification images of boxed areas in **a-a′**) (n = 4). *In situ* hybridization for *Cdh5* and *Lyve1* (**c,d**) or *Prox1* and *Lyve1* (**c′d′**) on a transverse vibratome section from an E10.5 embryo (**d-d′** - high magnification images of boxed areas in **c-c′**) (n = 4). (**e**) Partition-based graph abstraction (PAGA) inference of developmental trajectories on 19,699 cells. Cellular states were manually annotated based on known gene expression patterns. FA embedding showing Waddington-OT-based optimal transport analysis of (**f**) descendants of E9.5 venous cells, (**g**) descendants of E10.5 venous cells, (**h**) descendants of E9.5 angioblasts, (**i**) descendants of E10.5 angioblasts, (**j**) ancestors of E10.5 LEC progenitors and (**k**) ancestors of E10.5 LECs. (**l-m′**) Representative whole mount imaging of *Pecam1*, *Etv2* and *Prox1* expression at E9.0 using hybridization chain reaction (n = 6). (**n-o′**) Representative whole mount imaging of *Pecam1*, *Etv2* and *Prox1* expression at E10.0 using hybridization chain reaction (n = 6). (CV, cardinal vein; DA, dorsal aorta; He, heart; LB, liver bud; EC, endothelial cell; LEC, lymphatic endothelial cell; SV, sinus venosus; SHF, second heart field; PXM, paraxial mesoderm; LPM, lateral plate mesoderm; aSHF, anterior second heart field; pSHF, posterior second heart field; RBC, red blood cell; PGC, primordial germ cell; Scale bars - 100 μm (**a**-**a**′, **c**-**c**′), 50 μm (**b**-**b**′, **d**-**d**′), 1 mm (**l**-**o**′)).

To more precisely define molecular transitions during LEC differentiation, we performed single-cell RNA sequencing (scRNA-seq) at the onset of LEC specification (E9.5), during the emergence of LEC progenitors from venous endothelium (E10.5) and as primordial thoracic duct (pTD) formation begins (E11.5) (Fig. 1e)^18,19^. Due to the restricted contribution of the *Pax3* lineage to LECs of the trunk^20^, at each stage we dissected embryos at the level of the otic vesicle and first pharyngeal arch (Fig. 1e). To further enrich for cells of interest, at E9.5 VEGFR2^+^ cells were FACS-sorted with the goal of capturing ECs as well as any residual VEGFR2^+^ progenitors differentiating from mesoderm^21^ (Extended Data Fig. 2c). To maximize the diversity of EC subtypes collected at E10.5/E11.5, we captured cells that expressed VEGFR2 and/or PECAM1 (Extended Data Fig. 2d,e). A total of 19,699 high-quality cells were collected across three developmental stages (Extended Data Fig. 3a,b), with clustering of the merged dataset using Seurat version 4 revealing 30 distinct cellular states (Extended Data Fig. 3c,d). For visualization and to characterize putative transitions between cellular states, we used partition-based graph abstraction (PAGA) followed by ForceAtlas2 embedding (Fig. 1f). Each cellular state was comprised, to differing extents, of cells from each lineage (Fig. 1g and Extended Data Fig. 3d) and developmental stage (Fig. 1h and Extended Data Fig. 3d) and included paraxial mesoderm (*Pax3*^*+*^, *Lbx1*^*+*^, *Prrx2*^*+*^), angioblasts (*Etv2*^*+*^, *Tal1*^*+*^), venous ECs (*Cdh5*^*+*^, *Nr2f2*^+^, *Dab2*^+^), artery ECs (*Cdh5*^*+*^, *Gja5*^*+*^, *Bmx*^*+*^), LEC1 (*Prox1*^+^) and LEC2 (*Prox1*^+^, *Lyve1*^*+*^) (Fig. 1i and Extended Data Fig. 4a). Differential gene expression analyses between *Pax3* lineage-positive and lineage-negative ECs identified a number of genes with lineage-biased expression patterns (Extended Data Fig. 4b), including enhanced expression of genes linked to retinoic acid signaling (*Cyp26b1*, *Rbp1*, *Stra6*), a signaling pathway previously linked to lymphatic development^22,23^. Cluster identities were experimentally validated in E10.5 embryos using immunofluorescence and fluorescence in situ hybridization (Extended Data Fig. 5a–d′). We found that VWF (Extended Data Fig. 5a–b′), enriched in venous and arterial clusters (Fig. 1f and Extended Data Fig. 4a), was expressed throughout the dorsal aorta and CV but not by *Prox1*^+^ ECs in the surrounding mesenchyme. Expression of *Lyve1*, which was enriched in the venous, sinus venosus and liver EC clusters (Fig. 1f and Extended Data Fig. 4a), was restricted to the dorsal CV and not expressed by *Prox1*^+^ ECs in the surrounding mesenchyme at this stage (Extended Data Fig. 5c–d′).

### LECs arise directly from specialized mesenchymal progenitors

To gain further insight into the cell state transitions that occur during LEC specification, we used PAGA-based trajectory inference. These analyses revealed known developmental transitions within our dataset, including a trajectory from sinus venosus ECs to liver ECs^24^ (Extended Data Fig. 5e), and indicated that LECs can arise directly from angioblasts without transition through a venous EC state (Extended Data Fig. 5e). These findings were supported by Waddington Optimal Transport (Waddington-OT) (Extended Data Fig. 5f–k). In agreement with these findings, detailed examination of gene expression revealed an angioblast-like cellular state comprising cells that express mesenchymal markers, including *Prrx2* (ref. ^25^), the endothelial pioneer factor *Etv2* (ref. ^26^) and moderate expression of the master regulator of LEC fate *Prox1* (Fig. 1i). Furthermore, co-expression analyses identified single cells that express both *Etv2* and *Prox1* (Fig. 1j), which may represent bona fide lymphangioblasts. Collectively, these data suggest that LECs may arise directly from a dedicated pool of *Etv2*^*+*^*Prox1*^*+*^ angioblasts.

To characterize the spatiotemporal distribution of *Etv2*^*+*^*Prox1*^*+*^ cells during development, we performed hybridization chain reaction (HCR) and immunofluorescence on whole embryos and vibratome sections between E9.0 and E10.0 (Fig. 1k–m′ and Extended Data Fig. 5l–o′). Whole-mount imaging of *Etv2*, *Prox1* and *Pecam1* expression identified *Etv2*^*+*^*Prox1*^−^*Pecam1*^−^ angioblasts within the somitic paraxial mesoderm at E9.0 (Extended Data Fig. 5l–m′). By E9.5, *Etv2* expression was reduced in the anterior somites (Fig. 1k), where strong *Prox1* expression was observed anterior to the forelimb bud, as previously described^15^. High-resolution imaging of *Etv2* and *Prox1* co-expression revealed a population of *Etv2*^*+*^*Prox1*^+^*Pecam1*^–^ angioblasts emerging from the hypaxial dermomyotome at E9.5 (Fig. 1k′). These findings were confirmed with immunofluorescence imaging in *Pax3*^*Cre/+*^*;Rosa26*^*tdTomato*^ embryos, showing that paraxial mesoderm-derived, ETV2^+^PROX1^+^ angioblasts differentiate within mesenchyme surrounding the CV at E9.5 (Fig. 1l–l″). At E10, *Etv2*^+^ cells were largely absent from the lymphatic anlage anterior to the limb bud, highlighting the transient nature of this cellular state (Extended Data Fig. 5n–o′). Notably, analysis of *Etv2*, *Prox1* and *Pecam1* expression revealed the presence of *Etv2*^*+*^*Prox1*^+^*Pecam1*^–^ angioblasts in the pharyngeal mesoderm (Fig. 1m,m′), a known source of cardiac LECs^27,28^. Collectively, these analyses identify specialized angioblasts for LECs that arise directly from paraxial and pharyngeal mesoderm.

**Fig. 2:**
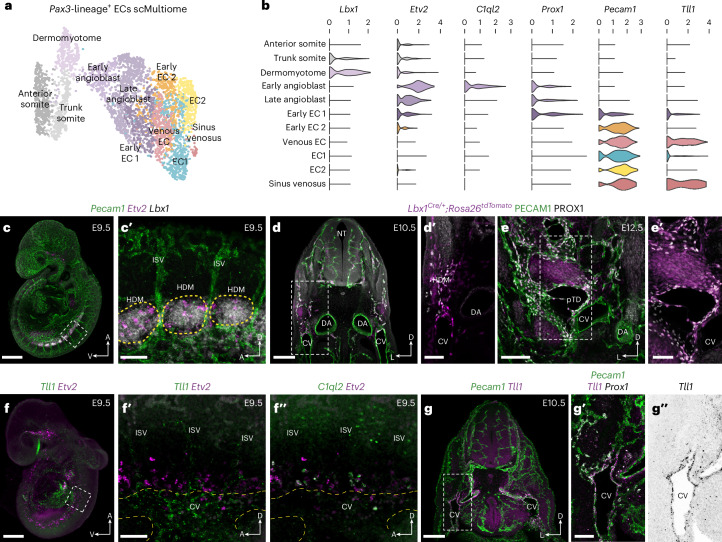
Multiomic analysis of paraxial mesoderm-derived endothelium. **a**, Weighted nearest neighbor UMAP (wnnUMAP) embedding of single-cell multiome analyses of 3,606 tdTomato^+^ VEGFR2^+^ PECAM1^+^ cells FACS-sorted from *Pax3*^*Cre/+*^*;Rosa26*^*tdtomato*^ embryos at E9.5. Cellular states were manually annotated based on known gene expression patterns and downstream analyses. **b**, Violin plots showing expression of selected genes across each cellular state. **c**, Representative whole-mount analysis of *Pecam1*, *Etv2* and *Lbx1* expression at E9.5 using HCR (*n* = 6). **c′**, High-magnification image of boxed area in **c** highlighting a population of *Etv2*^*+*^ angioblasts emerging from the *Lbx1*^*+*^ hypaxial dermomyotome. **d**,**d′**, Representative immunofluorescence for tdTomato, PECAM1 and PROX1 on a transverse vibratome section from an *Lbx1*^*Cre/+*^;*Rosa26*^*tdTomato*^ embryo at E10.5 (*n* = 4). **e**,**e′**, Representative immunofluorescence for tdTomato, PECAM1 and PROX1 on a transverse vibratome section from an *Lbx1*^*Cre/+*^;*Rosa26*^*tdTomato*^ embryo at E12.5 (*n* = 4). **f**, Representative whole-mount analysis of *Tll1* and *Etv2* expression at E9.5 using HCR (*n* = 6). High-magnification images of boxed area in **f** highlighting *Tll1* expression in CV ECs (**f′**) and *C1ql2* expression in a subset of *Etv2*^*+*^ angioblasts (**f**″). **g**, Representative HCR analysis of *Pecam1* and *Tll1* on a vibratome section from an E10.5 embryo. **g′**,**g**″, High-magnification images of boxed area in **g** showing *Pecam1*, *Tll1* and *Prox1* expression (*n* = 4). CV, cardinal vein; DA, dorsal aorta; HDM, hypaxial dermomyotome; ISV, intersegmental vessel; pTD, primordial thoracic duct. Scale bars, 500 μm (**c** and **f**), 100 μm (**c′**, **d′**, **e**, **f′** and **g′**), 200 μm (**d** and **g**), 50 μm (**e′**).

**Extended Data Fig. 6:**
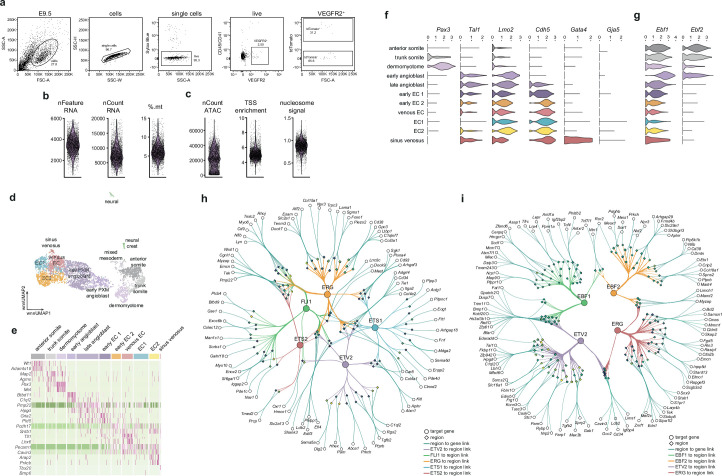
scMultiome Quality Control and gene regulatory network analysis. (**a**) Gating strategy to sort individual cells from *Pax3*^*Cre/+*^*;Rosa26*^*tdTomato*^ embryos for scRNA-seq at E9.5. (**b**) Quality control (QC) plots showing RNA features, counts and percentage of mitochondrial reads per cell. (**c**) Quality control (QC) plots showing ATAC counts, transcription start site (TSS) and percentage nucleosome signal per cell. (**d**) Weighted nearest neighbour Uniform Manifold Approximation and Projection (wnnUMAP) embedding of single cell multiome sequencing of 3,801 tdTomato^+^ VEGFR2^+^ PECAM1^+^ cells FACS-sorted from *Pax3*^*Cre/+*^*;Rosa26*^*tdtomato*^ embryos at E9.5. (**e**) Heatmap showing normalized expression of two diagnostic markers for each cell state on the subset of 3,606 cells in Fig. 2a. (**f**) Violin plots showing expression of *Pax3*, *Tal1*, *Lmo2*, *Cdh5*, *Gata4* and *Gja5* across each cellular state. (**g**) Violin plots showing expression of *Ebf1* and *Ebf2* across each cellular state. (**h**) Visualisation of the eGRN formed by ETV2, FLI1, ERG, ETS1 and ETS2. The network consists of selected transcription factors and the top 300 most variable regions and genes. (**i**) Visualisation of the eGRN formed by ETV2, ERG, EBF1 and EBF2. The network consists of selected transcription factors and the regions and genes with the 30 highest triplet scores for each of the chosen eRegulons.

To better characterize the molecular transitions associated with LEC specification from paraxial mesoderm, we performed single-cell Multiome (scMultiome) analyses of *Pax3* lineage-positive VEGFR2^+^ nuclei isolated from *Pax3*^*Cre/+*^*;Rosa26*^*tdTomato*^ embryos at E9.5 (Extended Data Fig. 6a). A total of 3,801 high-quality single cells were collected (Extended Data Fig. 6b–d), with clustering of the merged dataset using Seurat version 5 revealing 12 distinct cellular states (Extended Data Fig. 6d). To focus on cells of interest, we computationally removed the neural, neural crest and mixed mesoderm populations. Re-clustering of this subset of 3,606 cells revealed 11 cellular states (Fig. 2a,b and Extended Data Fig. 6e,f), including dermomyotome (*Lbx1*^*+*^, *Pax3*^*+*^), early angioblast (*Etv2*^*High*^, *C1ql2*^*+*^, *Tal1*^*+*^, *Lmo2*^*+*^), late angioblast (*Etv2*^*+*^, *Prox1*^*+*^, *Pecam1*^*−*^), early EC1 (*Prox1*^*+*^, *Pecam1*^*+*^), early EC2 (*Prox1*^*low*^, *Pecam1*^*High*^), venous ECs (*Tll1*^*+*^) and sinus venosus ECs (*Gata4*^*+*^, *Tll1*^*+*^). Expression of the artery marker *Gja5* suggests that there is no clear contribution of paraxial mesoderm-derived cells to arterial endothelium (Extended Data Fig. 6f), as previously observed in our scRNA-seq analyses across multiple stages (Extended Data Fig. 3d). We experimentally validated cluster identity and differentiation potential using HCR, immunofluorescence imaging and genetic lineage tracing (Fig. 2c–g″). Combined analysis of *Pecam1*, *Etv2* and *Lbx1* expression showed that *Etv2*^*+*^*Pecam1*^−^ angioblasts are specified within the *Lbx1*^+^ hypaxial domain of the dermomytome^29^ at E9.5 (Fig. 2c,c′). Immunofluorescence imaging of lineage-traced *Lbx1*^*Cre/+*^*;Rosa26*^*tdTomato*^ embryos showed tdTomato labeling of PROX1^+^ ECs in the dorsal portion of the CV and surrounding mesenchyme at E10.5 (Fig. 2d,d′) and the pTD and jugular lymph sac (JLS) at E12.5 (Fig. 2e,e′). Whole-mount imaging of *Tll1* and *Etv2* showed restricted expression of *Tll1* in the anterior portion of the CV as well as substantial labeling of the sinus venosus at E9.5 (Fig. 2f,f′). Limited expression of *Tll1* was also observed in venous intersegmental vessels but did not overlap with expression of *Etv2* (Fig. 2f′). Notably, imaging of *C1ql2* and *Etv2* revealed co-expression in a subset of angioblasts (Fig. 2f″), supporting our scMultiome analyses (Fig. 2a,b). Imaging of transverse sections from E10.5 embryos revealed restricted expression of *Tll1* in the CV endothelium (Fig. 2g), which was absent from *Prox1*^*+*^ ECs in the surrounding mesenchyme (Fig. 2g′,g″). *Tll1* expression was also observed in mural cells surrounding the dorsal aorta (Fig. 2g–g″). Collectively, these analyses validate our scMultiome sequencing, identify molecular differences between *Prox1*^*+*^ ECs found in the CV and those found in the surrounding mesenchyme and suggest that a considerable proportion of *Prox1*^*+*^ ECs are specified directly from angioblasts.

**Fig. 3:**
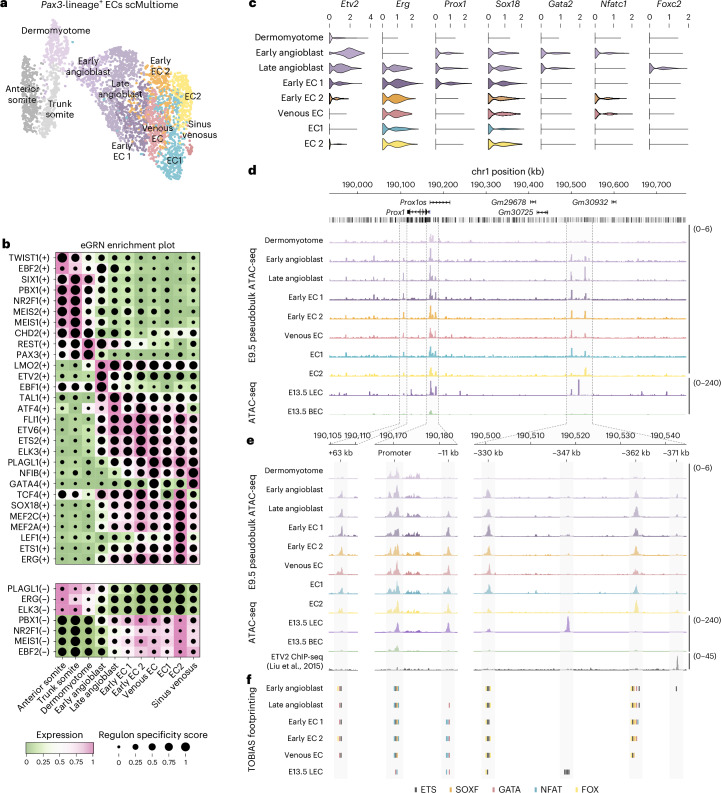
GRN analyses during LEC specification. **a**, Weighted nearest neighbor UMAP (wnnUMAP) embedding of single-cell multiome analyses of 3,606 tdTomato^+^ VEGFR2^+^ PECAM1^+^ cells FACS-sorted from *Pax3*^*Cre/+*^*;Rosa26*^*tdtomato*^ embryos at E9.5. **b**, Combined heatmap and dotplot showing TF expression of individual eRegulons and cell type specificity (regulon specificity score), respectively. Activation or repression of gene expression is indicated by (+) or (−). **c**, Violin plots showing expression of known transcriptional regulators of *Prox1* across each cellular state. **d**, Chromatin profiles at the *Prox1* genomic locus for E9.5 pseudobulk and E13.5 bulk ATAC-seq analyses. Pseudobulk profiles are derived from combining cells within each cellular state. **e**, Zoomed views of regions highlighted in **d**, including ETV2 ChIP-seq^37^. **f**, Putative binding of ETS, SOXF, GATA, NFAT and FOX TFs identified with TOBIAS^81^.

To investigate transcription factor (TF) activity and chromatin dynamics during LEC specification, we performed gene regulatory network (GRN) analyses and assessed chromatin composition and putative TF binding at the *Prox1* locus (Fig. 3a–f). We used SCENIC+ to identify enhancer-driven GRNs (eGRNs) comprising *cis*-regulatory elements, upstream TFs and candidate target genes (Fig. 3b and Extended Data Fig. 6h,i). These analyses highlighted the roles of ETS TFs during EC specification (ETV2; Fig. 3b and Extended Data Fig. 6h) and differentiation (FLI1, ETV6, ETS2, ELK3, ETS1, ERG; Fig. 3b and Extended Data Fig. 6h) and identified known regulators of EC gene expression (MEF2A, MEF2C; Fig. 3b) and LEC specification (SOX18; Fig. 3b)^30^. Additionally, eGRN analyses revealed putative regulons that may play roles in the differentiation of ECs and LECs (Fig. 3b**)**, including regulatory nodes driven by the early B cell factor family members EBF1 and EBF2 (Fig. 3b and Extended Data Fig. 6g,i). EBF1 is a pioneer factor required for B cell differentiation^31^ and neuronal development^32^, whereas EBF2 regulates bone formation^33^ and specification of brown adipose tissue^34^. Network analyses revealed *cis*-regulatory elements and target genes that may be co-regulated by ETV2 and EBF1/EBF2, indicating that these TFs may also act during EC specification (Extended Data Fig. 6i).

In a more targeted approach, we investigated regulation of *Prox1* (Fig. 3c–f). We characterized the expression of known regulators of *Prox1*, which have been studied at later stages of embryonic development or in mature LECs^30,35^, including *Sox18*, *Gata2*, *Nfatc1* and *Foxc2* (Fig. 3c). Notably, these TFs are expressed in angioblasts and, thus, may play similar roles in the initial induction of *Prox1* expression during LEC specification from angioblasts (Fig. 3c). To assess chromatin composition at the *Prox1* locus, we generated pseudobulk assay for transposase-accessible chromatin with sequencing (ATAC-seq) profiles by combining reads from individual cells within clusters and contrasted these with bulk ATAC-seq data from FACS-sorted LECs and BECs (Fig. 3d). These analyses identified a number of putative *cis-*regulatory elements that display increased accessibility during differentiation from dermomyotome to endothelium (Fig. 3d,e), including a previously described enhancer element that sits 11 kb upstream of the *Prox1* transcription start site (mm10:chr1:190,181,400–190,182,400 bp)^36^. Additionally, we identified elements at +63 kb (mm10:chr1:190,106,700–190,107,700), −330 kb (mm10:chr1:190,500,600–190,501,600), −347 kb (mm10:chr1:190,517,800-190,518,800), −362kb (mm10:chr1:190,532,650-190,533,650) and −371 kb (mm10:chr1:190,541,800–190,542,400), which are accessible at the onset of *Prox1* expression in angioblasts (Fig. 3c,e). To investigate regulation of *Prox1* by these putative *cis*-regulatory elements, we performed ATAC-seq footprinting using TOBIAS (Fig. 3f). TOBIAS is a digital genomic footprinting tool that maps Tn5 insertion events to characterize TF occupancy at known binding motifs. As individual motifs may be bound by multiple TFs from the same family in a manner that is indistinguishable to TOBIAS, we highlighted motifs bound by TF family members (for example, ETS, SOXF, GATA, NFAT, FOX) rather than binding by individual TFs (for example, ETV2, ERG, SOX18, GATA2, NFATC1, FOXC2) (Fig. 3f). These analyses suggest that described regulators of *Prox1* may bind to proximal promoter elements and *cis*-regulatory regions to drive *Prox1* expression in angioblasts and early ECs, displaying TF co-occupancy at several of these regulatory elements (Fig. 3f). As extensive binding of ETS TFs is predicted in angioblasts, we probed the possibility of direct regulation of *Prox1* by ETV2 by comparing our pseudobulk and bulk ATAC profiles with published ETV2 chromatin immunoprecipitation followed by sequencing (ChIP-seq) analyses in mouse embryonic stem cells^37^. These analyses identified an ETV2 ChIP-seq peak overlapping the accessible element at −371 kb, which was also predicted to be bound by ETS TFs in early angioblasts (Fig. 3f); it is possible that ETV2 also binds additional elements in the *Prox1* locus in angioblasts that are not bound in embryonic stem cells. Enhancer usage is known to be highly dynamic during embryonic development, with individual enhancers driving stage-specific biological functions^38^. Our data suggest dynamic remodeling of chromatin composition and TF binding within the *Prox1* locus during development, as we observed that regions of highly accessible chromatin in E9.5 angioblasts and ECs are closed in E13.5 LECs, whereas other regions display increased accessibility at later stages (Fig. 3d,e). Collectively, our analyses suggest that LEC specification from paraxial mesoderm is accompanied by chromatin remodeling and TF binding in angioblasts and implicate a number of known regulators of *Prox1* in this process.

### Temporal labeling of paraxial mesoderm derivatives

**Extended Data Fig. 7:**
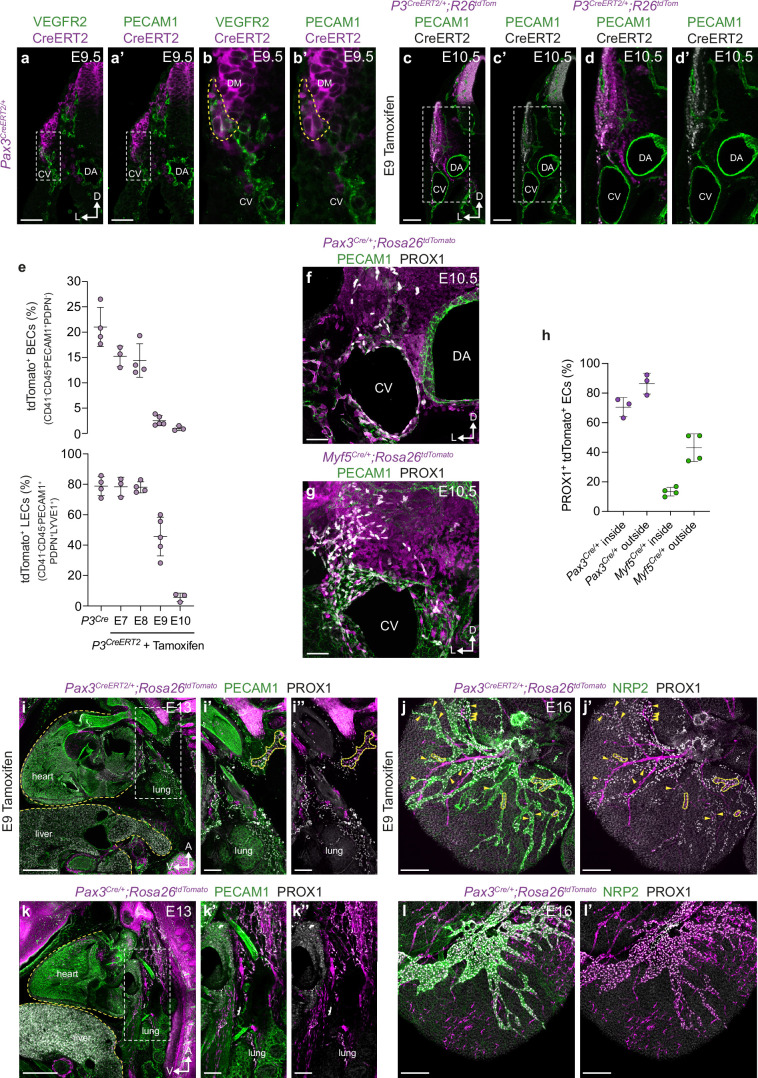
Spatiotemporal analyses of LEC lineage. Representative immunofluorescence image of VEGFR2 and CreERT2 (detected with an ESR1 antibody) (**a**, **b**) or PECAM1 and CreERT2 (**a′**, **b′**) on a transverse vibratome section from an E9.5 *Pax3*^*CreERT2/+*^ embryo (**b**-**b′** - high magnification image of boxed area in **a-a′**) (n = 4). (**c**-**c′**) Representative immunofluorescence image of tdTomato, PECAM1 and CreERT2 on a transverse vibratome section from a *Pax3*^*CreERT2/+*^*;Rosa26*^*tdTomato*^ embryo at E10.5 following tamoxifen administration at E9 (n = 4). (**d**-**d′**) High magnification image of boxed area in **c-c′**. (**e**) Quantification of tdTomato labelling of BECs and LECs by flow cytometry in *Pax3*^*Cre/+*^*;Rosa26*^*tdTomato*^ and *Pax3*^*CreERT2/+*^*;Rosa26*^*tdTomato*^ embryos at E13.5. Tamoxifen was administered to *Pax3*^*CreERT2/+*^*;Rosa26*^*tdTomato*^ animals at E7 (n = 3, 1 pregnant dam), E8.0 (n = 4, 2 pregnant dams), E9.0 (n = 5, 3 pregnant dams) or E10.0 (n = 3, 2 pregnant dams). Representative immunofluorescence images of tdTomato, PECAM1 and PROX1 on transverse vibratome sections from (**f**) *Pax3*^*Cre/+*^*;Rosa26*^*tdTomato*^ (n = 3) or (**g**) *Myf5*^*Cre/+*^*;Rosa26*^*tdTomato*^ (n = 4) embryos at E10.5. (**h**) Quantification of percentage tdTomato labelling of PROX1^+^ ECs present inside or outside of the venous endothelium in *Pax3*^*Cre/+*^*;Rosa26*^*tdTomato*^ or *Myf5*^*Cre/+*^*;Rosa26*^*tdTomato*^ embryos at E10.5. (**i**-**i**″) Representative immunofluorescence image of tdTomato, PECAM1 and PROX1 on a sagittal vibratome section from a *Pax3*^*CreERT2/+*^*;Rosa26*^*tdTomato*^ embryo at E13 following tamoxifen administration at E9 (n = 4). (**j**-**j′**) Representative immunofluorescence image of tdTomato, NRP2 and PROX1 on a whole mount heart from a *Pax3*^*CreERT2/+*^*;Rosa26*^*tdTomato*^ embryo at E16.0 following tamoxifen administration at E9 (n = 4). (**k-k**″) Representative immunofluorescence image of tdTomato, PECAM1 and PROX1 on a sagittal vibratome section from a *Pax3*^*Cre/+*^*;Rosa26*^*tdTomato*^ embryo (n = 4). (**l-l′**) Representative immunofluorescence image of tdTomato, NRP2 and PROX1 on a whole mount heart from a *Pax3*^*Cre/+*^*;Rosa26*^*tdTomato*^ embryo at E16.0 (n = 4). (DM, dermomyotome; CV, cardinal vein; DA, dorsal aorta; pTD, primordial thoracic duct; LS, lymph sac; Scale bars - 50 μm (**a**-**a**′, **f**, **g**), 100 μm (**c**-**c**′, **i**′-**i**″), 200 μm (**j**-**k**′), 500 μm (**i**); Data are presented as mean ± s.d. (error bars)). Source data

We next sought to assess the anatomical distribution of LECs derived from the paraxial mesoderm at distinct developmental stages using a tamoxifen-inducible *Pax3*^*CreERT2*^ driver^39^. For validation, we performed immunofluorescence with an ESR1 antibody to detect CreERT2. Strong CreERT2 expression was detected in the dorsal neural tube and dermomyotome at E9.5 (Extended Data Fig. 7a-b′) and E10.5 (Extended Data Fig. 7c-d′). Notably, in agreement with previous reports^21^, we found that *Pax3*-driven CreERT2 expression overlapped with VEGFR2 in the hypaxial dermomyotome at E9.5 (Extended Data Fig. 7a-b′) and was absent from PECAM1^+^ ECs at E9.5 and E10.5 (Extended Data Fig. 7a–d′), demonstrating the utility of this line for labeling paraxial mesoderm-derived ECs. To assess the timing of LEC specification from the paraxial mesoderm, we administered a single dose of tamoxifen to pregnant *Pax3*^*CreERT2/+*^*;Rosa26*^*tdTomato*^ animals at E7.0, E8.0, E9.0 or E10.0 to label cells from ~E7.25, ~E8.25, ~E9.25 or ~E10.25, respectively. Embryos were collected at E13.5 and analyzed by flow cytometry to compare BEC and LEC labeling with constitutive labeling in *Pax3*^*Cre/+*^*;Rosa26*^*tdTomato*^ animals (Extended Data Fig. 7e). These analyses revealed that, although labeling of BECs is very limited after tamoxifen administration beyond E8, significant labeling of LECs persists after induction at E9, indicating that the fate of paraxial mesoderm-derived angioblasts becomes restricted to LECs as development progresses.

**Fig. 4:**
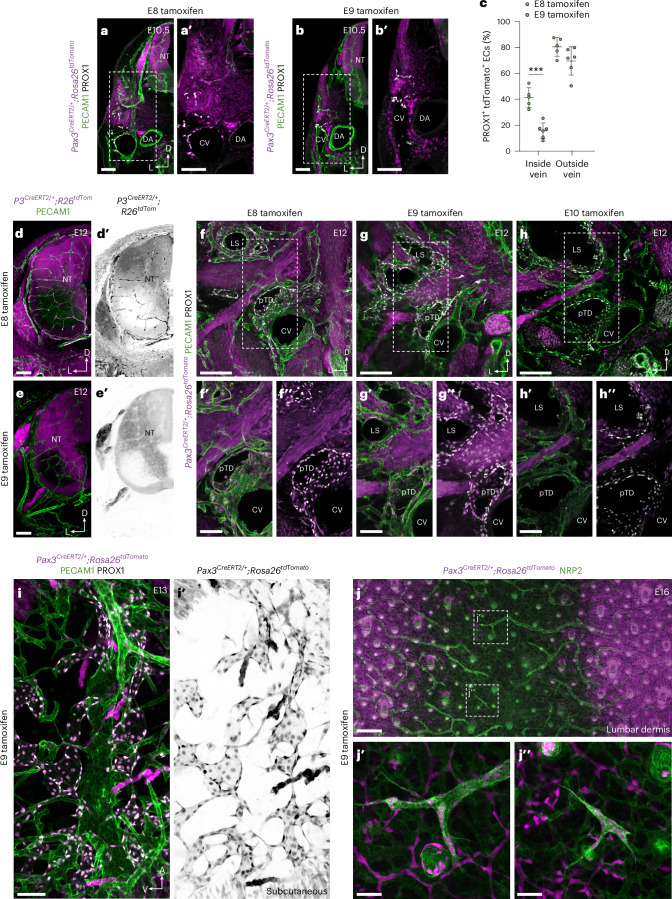
Temporal analysis of LEC specification from paraxial mesoderm. Representative immunofluorescence images of tdTomato, PECAM1 and PROX1 on transverse vibratome sections from *Pax3*^*CreERT2/+*^*;Rosa26*^*tdTomato*^ embryos at E10.5 after tamoxifen administration at E8 (**a**,**a′**) or E9 (**b**,**b′**). **c**, Quantification of percentage tdTomato labeling of PROX1^+^ ECs present inside or outside of the venous endothelium of *Pax3*^*CreERT2/+*^*;Rosa26*^*tdTomato*^ embryos at E10.5, after tamoxifen administration at E8.0 (*n* = 5, three pregnant dams) or E9.0 (*n* = 6, three pregnant dams). Each symbol represents data from an individual embryo (*** *P* < 0.001). Representative immunofluorescence images of tdTomato and PECAM1 on transverse vibratome sections from *Pax3*^*CreERT2/+*^*;Rosa26*^*tdTomato*^ embryos at E12.0 after tamoxifen administration at E8.0 (**d**,**d′**; *n* = 6) or E9.0 (**e**,**e′**; *n* = 6). Representative immunofluorescence images of tdTomato, PECAM1 and PROX1 on transverse vibratome sections from *Pax3*^*CreERT2/+*^*;Rosa26*^*tdTomato*^ embryos at E12.0 after tamoxifen administration at E8.0 (**f**–**f**″; *n* = 6), E9.0 (**g**–**g**″; *n* = 6) or E10.0 (**h**–**h**″; *n* = 6). **i**,**i′**, Representative immunofluorescence image of tdTomato, PECAM1 and PROX1 on a sagittal vibratome section from a *Pax3*^*CreERT2/+*^*;Rosa26*^*tdTomato*^ embryo at E13 after tamoxifen administration at E9 (*n* = 4). **j**–**j**″, Representative immunofluorescence images of tdTomato, NRP2 and PROX1 on whole-mount skin from *Pax3*^*CreERT2/+*^*;Rosa26*^*tdTomato*^ embryos after tamoxifen administration at E9 (*n* = 5). CV, cardinal vein; DA, dorsal aorta; LS, lymph sac; NT, neural tube; pTD, primordial thoracic duct. Scale bars, 100 μm (**a**,**a′**, **b**,**b′**, **d**,**d′**, **e**,**e′,** **f′**,**f″**, **g′**,**g″**, **h′**,**h″** and **i**,**i′**), 200 μm (**f**, **g** and **h**), 250 μm (**j**), 50 μm (**j′**,**j″**). Statistical analyses were performed using unpaired Student’s *t*-test. Data are presented as mean ± s.d. Source data

To investigate the spatial contribution of paraxial mesoderm derivatives to blood and lymphatic endothelium, we administered tamoxifen to *Pax3*^*CreERT2/+*^*;Rosa26*^*tdTomato*^ animals at multiple stages of development and performed immunofluorescence imaging (Fig. 4a–j″ and Extended Data Fig. 7i–j′). Imaging of transverse vibratome sections at E10.5 showed that most PROX1^+^ ECs in the mesenchyme surrounding the CV are labeled after tamoxifen administration at E8.0 (Fig. 4a,a′,c) or E9.0 (Fig. 4b,c). In contrast, labeling of PROX1^+^ venous ECs was significantly reduced after tamoxifen administration at E9.0 (Fig. 4b,c), with a limited proportion of PROX1^+^ ECs on the dorsal aspect of the CV labeled under these conditions (Fig. 4b,c). Of note, after tamoxifen administration at E9.0, we observed embryos in which a substantial proportion of PROX1^+^ ECs in the mesenchyme surrounding the CV were labeled in the absence of venous labeling, again suggesting that LEC specification can occur without transition through a venous intermediate. The observation that paraxial mesoderm-derived angioblasts continue to support venous expansion after E9.0 is in agreement with a recent study reporting labeled ECs in the CV after tamoxifen induction of *Etv2*^*CreERT2*^*;Rosa26*^*tdTomato*^ animals at E9.5 (ref. ^40^). Notably, using a Cre driver expressed from the *Myf5* locus (a myogenic transcription factor gene expressed from ~E9.0), we again found a contribution of labeled cells to the dorsal aspect of the CV at E10.5 (Extended Data Fig. 7f–h).

In agreement with our flow cytometry analysis of BEC and LEC labeling at E13.5 (Extended Data Fig. 7e), imaging of transverse vibratome sections at E12.0 showed a substantial contribution of *Pax3*-lineage cells to BECs in the neural tube after tamoxifen at E8.0 (Fig. 4d,d′), which was lost after E9.0 tamoxifen (Fig. 4e,e′). In contrast, imaging of transverse sections at E12.0 showed that most PROX1^+^ LECs in the pTD and lymph sac are labeled after tamoxifen administration at E8.0 (Fig. 4f–f″) or E9.0 (Fig. 4g–g″), whereas labeling is largely absent after tamoxifen administration at E10.0 (Fig. 4h–h″). These analyses show that, between E8.0 and E9.0, the fate of paraxial mesoderm-derived angioblasts becomes restricted to lymphatic endothelium and indicate that tamoxifen-independent tracing of LECs does not occur in *Pax3*^*CreERT2/+*^*;Rosa26*^*tdTomato*^ animals. After tamoxifen administration to *Pax3*^*CreERT2/+*^*;Rosa26*^*tdTomato*^ animals at E9, almost complete labeling of PROX1^+^ LECs was observed in subcutaneous tissues at E13 (Fig. 4i,i′) and in both lymphangiogenic sprouts (Fig. 4j,j′) and isolated LEC clusters (Fig. 4j,j″) of the lumbar dermis at E16; lymphatic vessels that were previously proposed to arise from a non-venous source^6^. In contrast, labeling of LECs in the heart at E13.0 (Extended Data Fig. 7i–i″) and E16.0 (Extended Data Fig. 7j,j′) was more limited than that observed in *Pax3*^*Cre/+*^*;Rosa26*^*tdTomato*^ embryos (Extended Data Fig. 7k–l′). Collectively, these analyses provide evidence for a specialized paraxial mesoderm-derived angioblast that is a major source of lymphatic endothelium.

### Reassessing the venous source of LECs

**Extended Data Fig. 8:**
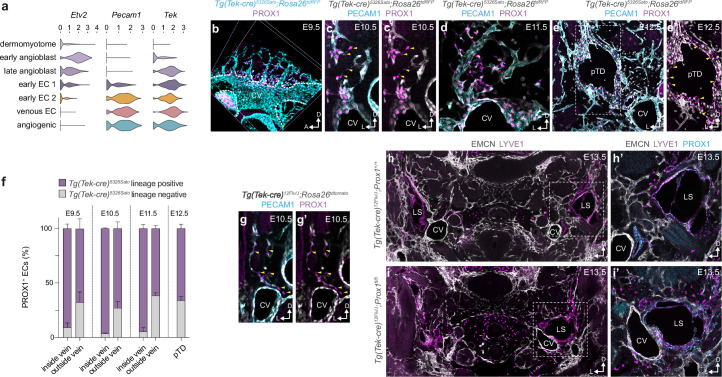
Reassessment of the venous origin of lymphatic endothelium. (**a**) Violin plots showing expression of *Tek* in angioblasts at E9.5. (**b**) Immunofluorescence for tdRFP and PROX1 on a whole mount *Tg(Tek-Cre)*^*5326Sato*^;*Rosa26*^*tdRFP*^ embryo at E9.5 (n = 3). Representative immunofluorescence images of tdRFP, PECAM1 and PROX1 on transverse vibratome sections from *Tg(Tek-Cre)*^*5326Sato*^;*Rosa26*^*tdRFP*^ embryos at (**c-c′**) E10.5, (**d**) E11.5 and (**e-e′**) E12.5 (n = 3). (**f**) Quantification of the percentage of lineage traced PROX1^+^ ECs in *Tg(Tek-Cre)*^*5326Sato*^;*Rosa26*^*tdRFP*^ embryos at E9.5-E12.5 (n = 3 per stage). (**g**-**g′**) Representative immunofluorescence image of tdTomato, PECAM1 and PROX1 from a transverse vibratome section of a *Tg(Tek-Cre)*^*12Flv/J*^;*Rosa26*^*tdRFP*^ embryo at E10.5 (n = 3). Immunofluorescence for EMCN, LYVE1 and PROX1 on transverse vibratome sections from (**h**-**h′**) *Tg(Tek-Cre)*^*12Flv/J*^;*Prox1*^*+/+*^ and (**i**-**i′**) *Tg(Tek-Cre)*^*12Flv/J*^;*Prox1*^*fl/fl*^ embryos at E13.5. (CV, cardinal vein; DA, dorsal aorta; pTD, primordial thoracic duct; LS, lymph sac; Scale bars - 25 μm (**c**-**c**′), 50 μm (**d**, **g**-**g**′), 200μm (**e**, **h**, **i**), 100μm (**h**′, **i**′); Data are presented as mean ± s.d. (error bars)). Source data

We next set out to re-evaluate the extent to which venous endothelium may serve as a source of mammalian LECs. Live imaging analyses in zebrafish embryos showed that LECs emerge from a subpopulation of venous ECs^7^, which undergo cell divisions that give rise to LECs and venous ECs^41^. The evidence for a predominantly venous source of LECs in mammals comes from lineage-tracing analyses of *Prox1*^*CreERT2*^ and *Tg(Tek-cre)* mice^9^. In contrast to our findings, which show concomitant initiation of PROX1 expression in the CV and surrounding mesenchyme at E9.5 (Fig. 1k′–l″), tamoxifen administration to *Prox1*^*CreERT2*^;*Rosa26*^*LacZ*^ animals at E9.5 led to sparse labeling of the dorsal CV and no labeling of non-venous cells at E13.5 (ref. ^9^), suggesting that early labeling of *Prox1*-expressing cells using this approach is inefficient. Analysis of our scRNA-seq dataset showed that *Tek* is expressed not only in venous ECs but also in angioblasts during LEC specification (Extended Data Fig. 8a). Furthermore, our previous analyses of dermal lymphatic vessel development in *Tg(Tek-cre)*^*12Flv/J*^*;Rosa26*^*mTmG*^ embryos identified double-reporter-positive LECs, indicative of recent recombination events induced by *Tg(Tek-cre)* in differentiated LECs^6^. Collectively, these observations bring into question the use of *Tg(Tek-cre)* mice as a tool for labeling only venous-derived LECs. Given these caveats, we used *Tg(Tek-cre)*^*5326Sato*^*;Rosa26*^*tdRFP*^ and *Tg(Tek-cre)*^*12Flv/J*^*;Rosa26*^*tdTomato*^ mice to revisit these analyses. Whole-mount immunofluorescence imaging of *Tg(Tek-cre)*^*5326Sato*^*;Rosa26*^*tdRFP*^ embryos revealed tdRFP^−^PROX1^+^ ECs dorsal to the CV at E9.5 (Extended Data Fig. 8b). Unlabeled PROX1^+^ ECs were also observed in vibratome sections of *Tg(Tek-cre)*^*5326Sato*^*;Rosa26*^*tdRFP*^ embryos at E10.5 (Extended Data Fig. 8c,c′) and E11.5 (Extended Data Fig. 8d) and in the pTD at E12.5 (Extended Data Fig. 8e,e′). Quantification of labeling at each stage revealed that approximately 30% of PROX1^+^ ECs outside of the venous endothelium are *Tg(Tek-cre)*^*5326Sato*^ lineage negative (Extended Data Fig. 8f), a figure that likely underestimates the true non-venous contribution given that *Tek* is expressed in angioblasts (Extended Data Fig. 8a). Similar observations were made in *Tg(Tek-cre)*^*12Flv/J*^*;Rosa26*^*tdTomato*^ embryos at E10.5 (Extended Data Fig. 8g,g′). Notably, the formation of lymph sac-like structures comprising LYVE1^+^PROX1^+^ LECs in *Tg(Tek-cre)*^*12Flv/J*^*;Prox1*^*fl/fl*^ embryos provides further evidence for a non-venous source of LECs (Extended Data Fig. 8h–i′). Collectively, these analyses provide evidence for a major non-venous contribution to developing lymphatics and raise questions about the use of *Tg(Tek-Cre)* mice as a tool to specifically label vein-derived LECs.

**Fig. 5:**
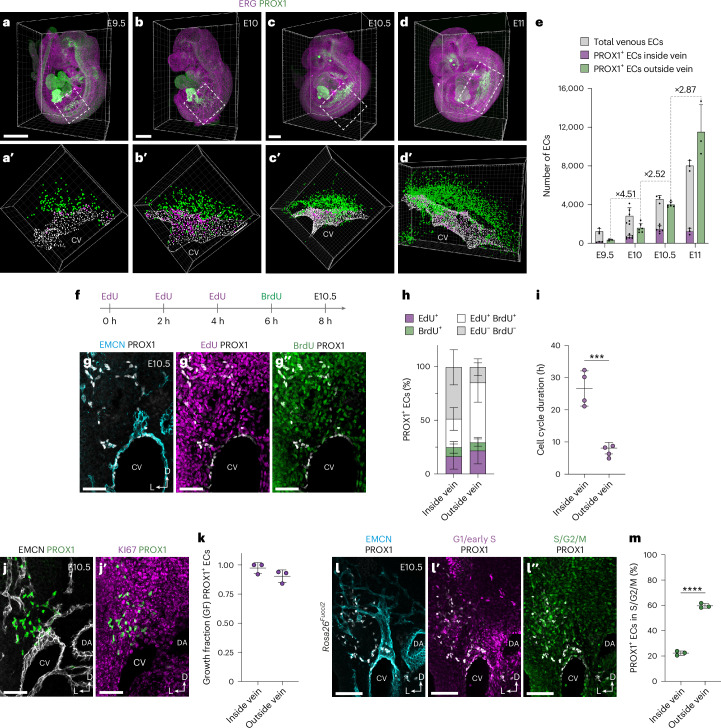
Quantitative analysis of LEC expansion. Representative whole-mount immunofluorescence for ERG and PROX1 in E9.5 (**a**,**a′**), E10 (**b**,**b′**), E10.5 (**c**,**c′**) and E11.0 (**d**,**d′**) embryos. 3D projections of regions of interest were segmented (**a′**–**d′**) for temporal quantification of ERG^+^PROX1^−^ ECs within the venous endothelium, ERG^+^PROX1^+^ ECs within the venous endothelium and ERG^+^PROX1^+^ ECs outside of the venous endothelium. **e**, Quantification of total ECs and PROX1^+^ ECs inside and outside of the venous endothelium between E9.5 and E11.0 (E9.5, *n* = 3; E10, *n* = 6; E10.5, *n* = 6; E11, *n* = 3). **f**, Schematic representation of the dual-pulse labeling strategy for analysis of cell cycle dynamics. **g**–**g**″, Representative immunofluorescence for EMCN, PROX1, EdU and BrdU on a transverse vibratome section from an E10.5 embryo. **h**, Quantification of labeling of PROX1^+^ ECs inside and outside of the venous endothelium with EdU and/or BrdU (*n* = 4). **i**, Quantification of cell cycle duration inside and outside of the venous endothelium at E10.5 (*n* = 4, *** *P* < 0.001). **j**,**j′**, Representative immunofluorescence for EMCN, PROX1 and KI67 on a transverse vibratome section from an E10.5 embryo. **k**, Quantification of growth fraction for PROX1^+^ ECs present inside or outside of the venous endothelium (*n* = 3). **l**–**l**″, Representative immunofluorescence for EMCN, PROX1, mCherry (G1/early S) and mVenus (S/G2/M) on a transverse vibratome section from an E10.5 *Rosa26*^*Fucci2*^ embryo. **m**, Quantification of PROX1^+^ ECs in S/G2/M phases of the cell cycle inside or outside of the venous endothelium (*n* = 3, **** *P* < 0.001). DA, dorsal aorta. Scale bars, 500 μm (**a**–**d**), 50 μm (**g**–**g″**), 100 μm (**j**,**j′** and **l**–**l″**). Statistical analyses were performed using unpaired Student’s *t*-test. Data are presented as mean ± s.d. Source data

**Extended Data Fig. 9:**
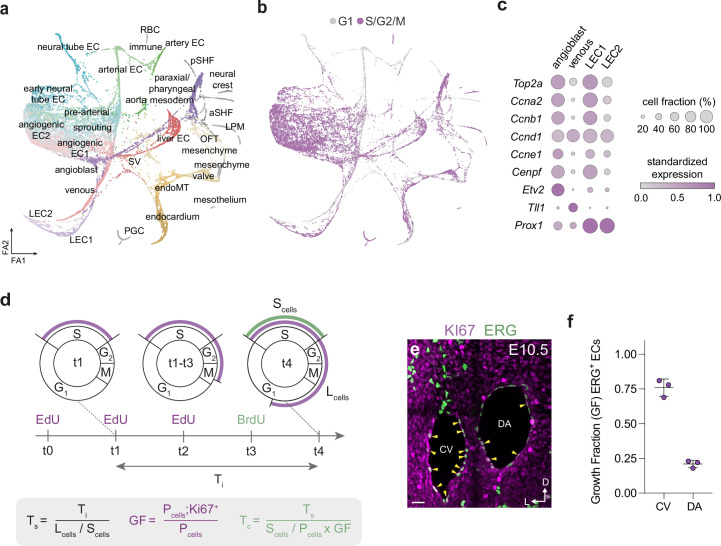
Analysis of LEC proliferation. (**a**) ForceAtlas2 (FA) embedding of 19,699 cells based on partition-based graph abstraction (PAGA), with each dot representing a single cell. (**b**) FA embedding showing cell cycle phase in each cellular state. (**c**) Expression of selected cell cycle-related genes in single cells in angioblast, venous, LEC progenitor and lymphatic states. (**d**) Dual-pulse labelling strategy for analysis of cell proliferation. (**e**) Representative immunofluorescence image of ERG and KI67 expression in a transverse vibratome section from an E10.5 embryo (n = 3). (**f**) Quantification of growth fraction for ECs present inside the CV or DA. (CV, cardinal vein; DA, dorsal aorta; Scale bars - 25 μm (d); Data are presented as mean ± s.d. (error bars)). Source data

To further evaluate the developmental source of LECs, we assessed cell proliferation, reasoning that if migrating venous ECs are the major source of LECs, a substantial increase in EC numbers would be required to maintain venous integrity during the rapid expansion of lymphatics that occurs from E10. Characterization of cell cycle phase (Extended Data Fig. 9a,b) and gene expression (Extended Data Fig. 9c) in our scRNA-seq dataset revealed that most cells in the venous cluster are in G1 (Extended Data Fig. 9a,b) and express low levels of genes associated with cell cycle progression (Extended Data Fig. 9c). In contrast, most cells in the angioblast and LEC1 clusters are in S/G2/M (Extended Data Fig. 9a–c). These analyses suggest that angioblast-derived LECs and their ancestors, and not venous ECs, are endowed with the proliferative capacity to support rapid growth of LECs. To assess the expansion of venous ECs, and PROX1^+^ ECs inside and outside of the veins, we performed quantitative whole-mount light sheet imaging of embryos stained for ERG and PROX1 between E9.5 and E11.0 (Fig. 5a–e). These analyses revealed a rapid expansion of PROX1^+^ ECs outside the vein, increasing approximately 32-fold between E9.5 and E11, from 351 ± 85 to 11,510 ± 2,832 cells (±s.d.) (Fig. 5a–e). In contrast, more modest increases were observed in total venous ECs and PROX1^+^ venous ECs, increasing approximaterly 6.5-fold (1,250 ± 301 to 8,025 ± 557 cells (±s.d.)) and approximately nine-fold (140 ± 32 to 1,242 ± 328 cells (±s.d.)), respectively (Fig. 5a–e).

To empirically assess the proliferation of PROX1^+^ ECs inside and outside of the venous endothelium, we used a dual-pulse labeling strategy (Fig. 5f–i and Extended Data Fig. 9d). To maintain ethynyl-2′-deoxyuridine (EdU) bioavailability over the course of the experiment, we administered EdU three times at 2-h intervals, followed by a 2-h 5-bromo-2′-deoxyuridine (BrdU) pulse (Fig. 5f). The growth fraction of PROX1^+^ ECs was calculated using KI67 immunofluorescence to account for potential differences in growth state between cells inside and outside the vein (Fig. 5j,k and Extended Data Fig. 9d) and contrasted with growth fraction in the entire CV or dorsal aorta (Extended Data Fig. 9e,f). Analysis of EdU and BrdU labeling revealed higher incorporation in PROX1^+^ ECs outside of the vein at E10.5 (Fig. 5g,h), which translated to significantly shorter cell cycle duration in these cells (26.6 ± 5.5 h inside vein and 8.1 ± 1.8 h outside vein (±s.d.); Fig. 5i). In agreement with these findings, analysis of *Rosa26*^*Fucci2*^ embryos^42^, where cells in G1/early S-phase are labeled with mCherry and cells in S/G2/M are labeled with mVenus, revealed that a higher proportion of PROX1^+^ ECs outside of the vein are in S/G2/M phase at E10.5 (Fig. 5l,m). Collectively, these findings show that rapid expansion of PROX1^+^ ECs outside of the vein (Fig. 5a–e) occurs despite only modest venous proliferation. Given the limited proliferation of venous endothelium at these stages, CV expansion may be supported by continued addition of cells from the paraxial mesoderm, as indicated by our lineage-tracing analyses (Fig. 4b,c). This model further challenges the prevailing view that, upon induction of PROX1 expression in venous ECs, LEC progenitors migrate dorsally from venous endothelium to form the first lymphatic structures.

**Fig. 6:**
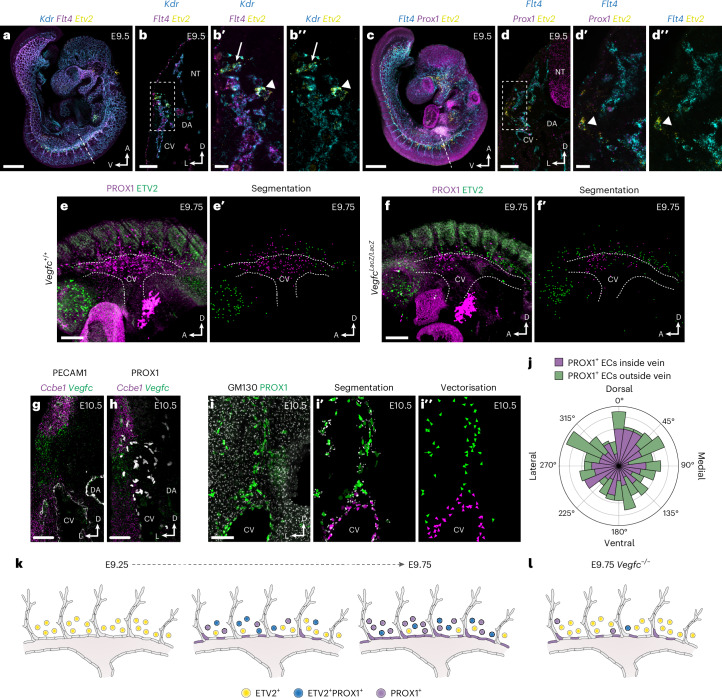
Temporal analyses of LEC morphogenesis. **a**, Representative confocal image of *Kdr*, *Flt4* and *Etv2* expression in a whole-mount E9.5 embryo using HCR (*n* = 6). **b**, Transverse vibratome section showing *Kdr*, *Flt4* and *Etv2* expression at the level of dashed line in **a** (*n* = 4). **b′**,**b**″, High-magnification images of boxed area in **b** showing *Kdr*, *Flt4* and *Etv2* expression. Arrow indicates a *Kdr*^+^ cell, arrowhead indicates a *Kdr*^+^*Etv2*^+^ cell. **c**, Representative confocal image of *Flt4, Prox1* and *Etv2* expression in a whole-mount E9.5 embryo using HCR (*n* = 6). **d**, Transverse vibratome section showing *Flt4*, *Prox1* and *Etv2* expression at the level of dashed line in **c** (*n* = 4). **d′**,**d**″, High-magnification images of boxed area in **d** showing *Flt4*, *Prox1* and *Etv2* expression. Arrowhead indicates a *Flt4*^+^*Prox1*^+^*Etv2*^+^ cell. Representative whole-mount immunofluorescence for PROX1 and ETV2 and image segmentation in *Vegfc*^*+/+*^ (**e**,**e′**) and *Vegfc*^*LacZ/LacZ*^ (**f**,**f′**) embryos at E9.75 (*n* = 3). Combined in situ hybridization for *Ccbe1* and *Vegfc* and immunofluorescence for PECAM1 (**g**) or PROX1 (**h**). **i**, Immunofluorescence for GM130 and PROX1 on a transverse vibratome section from an E10.5 embryo. Segmentation of GM130^+^ Golgi and PROX1^+^ EC nuclei (**i′**) and vectorization of Golgi orientation for quantification (*n* = 8) (**i**″). **j**, Rose diagram illustrating Golgi orientation in PROX1^+^ ECs inside and outside of the venous endothelium. Proposed model of LEC differentiation through in situ specification from ETV2^+^PROX1^+^ paraxial mesoderm-derived lymphangioblasts in WT (**k**) and *Vegfc*^*−/−*^ (**l**) embryos; colored circles represent individual cells expressing the indicated genes. DA, dorsal aorta; NT, neural tube. Scale bars, 500 μm (**a** and **c**), 100 μm (**b**, **d**, **g** and **h**), 25 μm (**b′** and **d′**), 200 μm (**e** and **f**), 50 μm (**i**).

**Extended Data Fig. 10:**
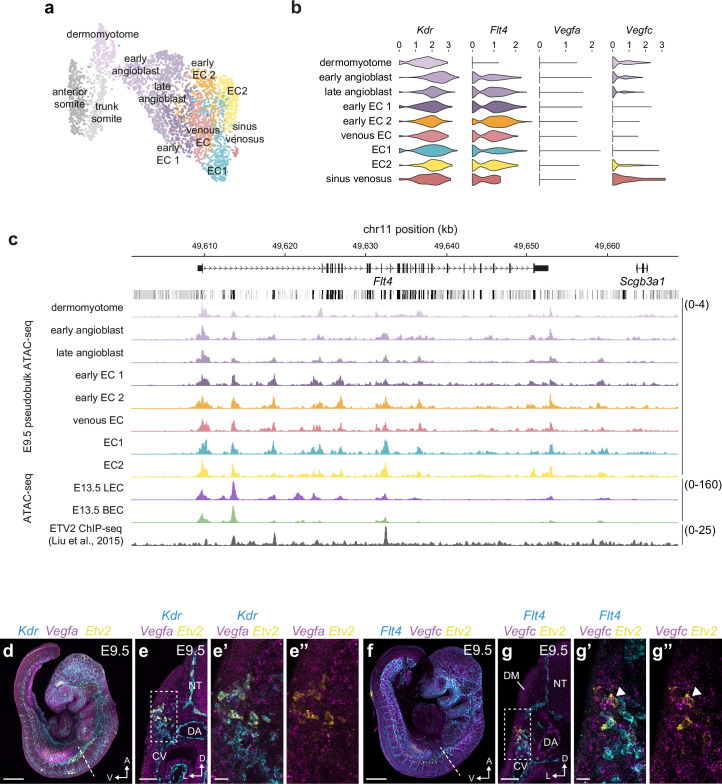
Expression and regulation of Vegf ligands and receptors. (**a**) Weighted nearest neighbour Uniform Manifold Approximation and Projection (wnnUMAP) embedding of single cell multiome analyses of 3,606 tdTomato^+^ VEGFR2^+^ PECAM1^+^ cells FACS-sorted from *Pax3*^*Cre/+*^*;Rosa26*^*tdtomato*^ embryos at E9.5. (**b**) Violin plots showing expression of Vegf ligands and receptors across each cellular state. (**c**) Chromatin profiles at the *Flt4* genomic locus for E9.5 pseudobulk and E13.5 bulk ATAC-seq analyses. (**d**) Representative confocal image of *Kdr*, *Vegfa* and *Etv2* expression in a whole mount E9.5 embryo using hybridization chain reaction (n = 6). (**e**) Transverse vibratome section showing *Kdr*, *Vegfa* and *Etv2* expression at the level of dashed line in **d** (n = 4). (**e′**-**e**″) High magnification images of boxed area in **f** showing *Kdr*, *Vegfa* and *Etv2* expression. (**f**) Representative confocal image of *Flt4, Vegfc* and *Etv2* expression in a whole mount E9.5 embryo using hybridization chain reaction (n = 6). (**g**) Transverse vibratome section showing *Flt4, Vegfc* and *Etv2* expression at the level of dashed line in **f** (n = 4). (**g′**-**g**″) High magnification images of boxed area in **g** showing *Flt4, Vegfc* and *Etv2* expression. Arrowhead highlighting *Etv2*/*Vegfc* double positive cell. (CV, cardinal vein; DA, dorsal aorta; NT, neural tube; DM, dermomyotome. Scale bars - 500 μm (**c**, **e**), 100 μm (**d**, **f**), 25 μm (**d**′, **f**′).

The migration of LECs is highly dependent on vascular endothelial growth factor-C (VEGFC)^19,43^; however, the role of VEGF signaling during LEC specification from paraxial mesoderm is unknown. Our scMultiome analyses indicate that *Etv2*^+^ angioblasts are initially specified from *Kdr*^*+*^ (*Vegfr2*) mesoderm and then sequentially upregulate *Flt4* (*Vegfr3*) and *Prox1* (Fig. 2a,b and Extended Data Fig. 10b). Interestingly, although we did not observe substantial expression of *Vegfa* (Extended Data Fig. 10b), *Vegfc* expression was observed in dermomyotome and angioblasts as well as in sinus venosus and neural tube ECs (Extended Data Fig. 10b). To validate these observations, we performed HCR analyses in whole-mount embryos at E9.5 (Fig. 6a–d″ and Extended Data Fig. 10d–g″). Combined analysis of *Kdr*, *Flt4* and *Etv2* expression revealed that all *Etv2*^*+*^ cells were *Kdr*^*+*^, and some *Etv2*^*+*^*Kdr*^*+*^ cells also expressed *Flt4* (Fig. 6a-b″), indicating that *Etv2* and *Kdr* co-expression precedes *Flt4* expression. Etv2 was shown to directly regulate *Flt4* expression in zebrafish embryos^44^. To investigate regulation of *Flt4* by ETV2 in mice, we compared our pseudobulk and bulk ATAC profiles with published ETV2 ChIP-seq^37^ and identified multiple overlapping peaks in the *Flt4* locus (Extended Data Fig. 10c), suggesting that ETV2 may bind these putative regulatory elements in angioblasts to drive chromatin remodeling or induce *Flt4* expression. FLT4 is required to maintain PROX1 expression in early LECs^45^, and our analysis of *Flt4*, *Prox1* and *Etv2* expression revealed that *Etv2*^*+*^*Prox1*^*+*^ cells always expressed *Flt4* (Fig. 6c,d″), suggesting that FLT4 may act upstream of PROX1 during angioblast differentiation. HCR analysis of *Vegf* ligands demonstrated broad, largely unrestricted *Vegfa* expression (Extended Data Fig. 10d-e″). In contrast, *Vegfc* expression was enriched in the dermomyotome and dorsal aorta and in *Etv2*^+^ angioblasts surrounding the CV (Extended Data Fig. 10f-g″), suggesting that it may act through both paracrine and autocrine signaling during LEC specification. Collectively, these findings suggest that VEGFC/VEGFR3 signaling acts upstream of PROX1 during LEC specification from paraxial mesoderm-derived angioblasts.

To address the functional importance of VEGFC signaling, we performed whole-mount immunofluorescence for PROX1 and ETV2 in *Vegfc*^*+/+*^ and *Vegfc*^*LacZ/LacZ*^ embryos (Fig. 6e-f′). As previously reported^43^, these analyses revealed a reduction in PROX1-expressing cells surrounding the CV (Fig. 6e-f′). Notably, this reduction was coupled to an increase in ETV2-expressing cells (Fig. 6e-f′), suggesting that, in the absence of VEGFC/VEGFR3 signaling, specification of LECs from paraxial mesoderm stalls at the level of the angioblast. These findings indicate that, rather than stimulating migration of ECs from venous endothelium into the surrounding mesenchyme, the primary function of VEGFC may be to drive in situ differentiation of angioblasts into PROX1^+^ ECs. To further assess the plausibility of PROX1^+^ EC migration from the CV, we combined in situ hybridization and immunofluorescence for *Ccbe1*, *Vegfc*, PECAM1 and PROX1 at E10.5 (Fig. 6g,h). These analyses revealed that *Vegfc* is expressed along the lateral body wall and that co-expression with *Ccbe1*, which is essential for processing of VEGFC into its lymphangiogenic form^46^, occurs in two domains (Fig. 6g,h). One domain sits dorsal to the CV, in the region of the hypaxial dermomyotome, and a second domain immediately flanks the ventrolateral CV. The absence of *Ccbe1* from the mesenchyme immediately dorsal to the CV, as well as the failure of venous PROX1^+^ ECs to enter the ventrolateral VEGFC/CCBE1 expression domain, suggests that paraxial mesoderm-derived angioblasts are more likely to be responsive to VEGFC/CCBE1 activity than venous PROX1^+^ ECs. To assess the direction of PROX1^+^ EC migration, we analyzed vibratome sections from E10.5 embryos stained for PROX1 and the Golgi marker GM130 (Fig. 6i–i″). During cell migration, the Golgi apparatus is positioned ahead of the nucleus in the direction of movement and can, thus, be used to infer directionality of migration^47^. Segmentation and vectorization of PROX1^+^ EC nuclei and their Golgi allowed quantification of Golgi orientation in the dorsal-ventral and lateral-medial axes, revealing that migration of PROX1^+^ ECs inside and outside of the vein is randomized (Fig. 6j). Collectively, our analyses challenge the view that venous endothelium is the primary source of LECs. Instead, we show that VEGFC induces in situ differentiation of LECs from a population of specialized angioblasts, which, in turn, give rise to a majority lymphatic endothelium.

## Discussion

At the beginning of the 20th century, Florence Sabin’s anatomical studies of pig^8^ and human^48^ embryos suggested that lymphatic vessels form through budding from large veins. An alternative model of LEC specification, directly from mesenchymal precursors, was later proposed by Huntington and McClure^5^. Debate over these two opposing models of LEC specification has continued for over a century, with evidence from a range of model systems suggesting the existence of venous, non-venous and dual sources^6,10,11,16,17,28,49^. In mammalian embryos, the consensus was that LECs arise predominantly through centrifugal sprouting from venous endothelium, with non-venous sources making limited contributions to organ-specific lymphatic networks^1,6,17^. Here, using a range of single-cell genomics, lineage tracing and high-resolution imaging, we show that LECs are specified in situ from specialized mesenchymal progenitors (Fig. 6k,l). Collectively, these analyses provide evidence for a specialized paraxial mesoderm-derived angioblast that is a major source of lymphatic endothelium at E13.5.

The term ‘specialized angioblast’ was previously used to describe a population of ECs in the zebrafish CV that give rise to arterial, venous and lymphatic endothelium^7^. Here, we identify bona fide ETV2^+^ mesenchymal angioblasts that do not express markers of mature endothelium, are not exposed to blood flow and directly give rise to PROX1^+^ ECs. Due to the transient nature of ETV2 and PROX1 co-expression, we were unable to determine if all angioblast-derived LECs co-express these two genes during their differentiation. However, our analyses show that transition from ETV2^+^ angioblast to PROX1^+^ EC is rapid. Notably, our single-cell analyses revealed not only that specification of LECs from *Prox1*^+^ venous endothelium is limited but also that paraxial mesoderm-derived angioblasts continue to contribute to venous expansion, observations supported by lineage-tracing and morphometric analyses. Indeed, given the limited proliferative capacity of venous endothelium, if all PROX1^+^ ECs were to migrate from the CV and sinus venosus, these vessels would cease to exist. Instead, our analyses show that there is a steady increase in venous EC numbers at this stage, collectively supporting a model of limited PROX1^+^ EC migration from venous endothelium. Ultimately, technological advances that permit the live imaging of mammalian embryos, at currently infeasible stages of development, may provide definitive evidence for venous or non-venous sources of LECs.

Mechanistically, we provide insight into the acquisition of LEC identity through global GRN analyses and examination of *cis*-regulatory elements in the *Prox1* locus. In agreement with the well-described roles of ETS factors in directing^26,50^ and maintaining^51^ EC fate, our analyses identified multiple eGRNs regulated by ETS TFs, including ETV2 and ERG. Interestingly, we also identified a number of putative regulators of LEC specification, including EBF1 and EBF2. EBF1 is a pioneer factor essential for the acquisition of B cell identity^52^ and heart development^53^, which can bind and remodel naive chromatin^52^. Although no obvious vascular defects have been described after loss of either *Ebf1* or *Ebf2*, EBF1 has been shown to functionally compensate for the loss of *Ebf2* in mouse adipocytes^54^. Thus, it is tempting to speculate that these factors may also contribute to establishment of a chromatin landscape permissive to LEC specification. Although the importance of PROX1 activity for acquisition and maintenance of LEC fate is well established, knowledge of the factors controlling its transcription is relatively limited. Detailed characterization of the *Prox1* −11-kb enhancer, which is bound by GATA2, FOXC2, NFATC1 and PROX1, recently revealed a role for this element in the repression of hemogenic fate in LECs^36^. Furthermore, elegant work in zebrafish has shown that multiple *cis*-regulatory elements are required to drive anatomically distinct patterns of *prox1a* expression during embryonic and larval development^55^. Here, our scMultiome analyses uncovered multiple putative enhancers of *Prox1* open in angioblasts and predicted to be bound by known transcriptional regulators. In future work, it will be of interest to investigate the spatiotemporal control of LEC specification and *Prox1* expression by the TFs and *cis*-regulatory elements identified here.

Our analyses suggest that, in addition to important roles in control of LEC migration^43,56^ and maintenance of LEC identity^57^, VEGFC/VEGFR3 is critical for the transition of differentiating angioblasts into PROX1^+^ ECs. During the process of LEC specification from paraxial mesoderm, VEGFR2 and ETV2 are expressed before VEGFR3. Given the key roles of VEGFR2 in angioblast specification at earlier stages of development, it is likely that initial specification of ETV2^+^ angioblasts in the hypaxial dermomyotome is VEGFR2 dependent, with subsequent differentiation of VEGFR2^+^ETV2^+^ angioblasts into PROX1^+^ ECs requiring VEGFC/VEGFR3. Our revised model of mammalian lymphatic vessel development suggests that, rather than sprouting from a contiguous venous-derived structure, expansion of the initial lymphatic network occurs through coalescence of extravascular PROX1^+^ ECs. This process resembles formation of lymphatic vessels from LEC clusters, which has been observed in the mesentery^17^, dermis^6^, heart^58^ and kidney^59^, and raises the possibility that the coalescence of clustered LECs is a more general mechanism for expansion of lymphatic networks throughout development.

The identification of angioblasts that directly give rise to a subset of mammalian endothelium has broad implications for our understanding of vascular development. Subtypes of angioblasts that form arterial, venous and intestinal ECs have been described in zebrafish embryos^7,12,40^. Furthermore, chimeric transplantation in avian embryos demonstrated the restricted potential of dermomyotome-derived cells, which were found to contribute to endothelium of the body wall and kidney, but did not integrate into endothelium of the visceral organs or the ventral wall of the dorsal aorta^60^. Based on our findings, it is tempting to speculate that further aspects of EC fate and function are underpinned by differences established at the level of the angioblast source from which they arise and before they acquire endothelial identity. An improved understanding of the determinants of BEC and LEC fate and function will shed further light on these processes, enhancing understanding of congenital and acquired vascular diseases and aiding attempts to engineer bona fide organ- and system-specific ECs in vitro.

## Methods

### Animal experiments

All procedures were carried out in accordance with local legislation: University of Oxford Animal Welfare and Ethical Review Boards in accordance with Animals (Scientific Procedures) Act 1986 under Home Office project licences PPL PC013B246 or PP6588077; German animal protection legislation (Tierschutzgesetz und Tierschutz-versuchstierverordnung); and Uppsala Animal Experiment Ethics Board (permit number 130/15).

### Mouse strains, husbandry and embryo collection

The following mice lines were used in this study: *Pax3*^*Cre*^ (*Pax3*^*tm1(cre)Joe*^)^61^; *Pax3*^*CreERT2*^ (*Pax3*^*tm1.1(cre/ERT2)Lepr*^)^39^; *Lbx1*^*Cre*^ (*Lbx1*^*tm3.1(cre)Cbm*^)^62^; *Myf5*^*Cre*^ (*Myf5*^*tm3(cre)Sor*^)^63^; Tie2-Cre (Tg(Tek-cre)^12Flv/J^)^64^; Tie2-Cre (Tg(Tek-cre)^5326Sato^)^65^; *Prox1*^*fl*^ (*Prox1*^*tm1a(EUCOMM)Wtsi*^)^6^; *Rosa26*^*tdTomato*^ (*Gt(ROSA)26Sor*^*tm9(CAG-tdTomato)Hze*^)^66^; *Rosa26*^*tdTomato*^ (*Gt(ROSA)26Sor*^*tm14(CAG-tdTomato)Hze*^)^66^; *Rosa26*^*tdRFP*^ (*Gt(ROSA)26Sor*^*tm1Hjf*^)^67^; *Rosa26*^*Fucci2*^ (Tg(Gt(ROSA)26Sor-Fucci2)^Sia^)^42^; and *Vegfc*^*LacZ*^ (*Vegfc*^*tm1Ali*^)^43^. With the exception of the *Vegfc*^*tm1Ali*^ strain, which was maintained on a CD1 background, all animals were maintained on a C57Bl/6 background. Mice were maintained in individually ventilated cages (IVCs) and ventilated racks at 22 °C and 55% humidity. For embryo collection, mice were paired overnight, and females were checked the next morning for the presence of a vaginal plug. For inducible Cre induction, pregnant females were gavaged at the specified timepoints with 80 mg kg^−1^ tamoxifen (Sigma-Aldrich, T5648) dissolved in peanut oil with 10% ethanol at a final concentration of 10 mg ml^−1^.

### Immunofluorescence staining of embryonic sections, whole-mount embryos and tissues

The following antibodies were used for immunofluorescence staining of cryosections, vibratome sections and/or whole-mount tissues: ETV2 (Abcam, ab181847, 1:100), VEGFR2 (BD Pharmingen, 550549, 1:200), PECAM1 (R&D Systems, AF3628, 1:250), PECAM1 (D. Vestweber, clones 5D2.6 and 1G5.1, 15 μg ml^−1^), PROX1 (R&D Systems, AF2727, 1:200), PROX1 (Proteintech, 11067-2-AP, 1:100), PROX1 (Reliatech, 102-PA32, 1:100), PROX1 (Abcam, ab225414, 1:100), EMCN (Santa Cruz Biotechnology, sc-65495, 1:50), EMCN (D. Vestweber, VE44, 1:100), EMCN (Santa Cruz Biotechnology, sc-53941, 1:50), VWF (Abcam, 11713, 1:100), ESR1 (Abcam, ab16660, 1:100), NRP2 (R&D Systems, AF567, 1:250), ERG (Abcam, ab92513, 1:200), GM130 (BD Pharmingen, 610823, 1:100), RFP (Rockland, 600-401-379, 1:500), BrdU (Abcam, ab6326, 1:100), KI67 (Thermo Fisher Scientific, 14-5698-82, 1:100) and GFP (Thermo Fisher Scientific, A-21311, 1:100). The following Alexa Fluor–conjugated secondary antibodies (Thermo Fisher Scientific) were used at 1:500–1:1,000: donkey anti-rat IgG Alexa Fluor Plus 405 (A48268), donkey anti-rabbit IgG Alexa Fluor 488 (A21206), donkey anti-goat IgG Alexa Fluor Plus 488 (A32814), donkey anti-sheep IgG Alexa Fluor 488 (A11015), donkey anti-rabbit IgG Alexa Fluor 555 (A32814), donkey anti-rat IgG Alexa Fluor 555 (A48270), donkey anti-goat IgG Alexa Fluor 647 (A32849), donkey anti-rabbit IgG Alexa Fluor Plus 647 (A32795) and donkey anti-sheep IgG Alexa Fluor 647 (A21448).

For immunofluorescence staining of cryosections, samples were fixed in 4% paraformaldehyde (PFA) overnight at 4 °C. Samples were washed in 1× PBS and then cryoprotected in sucrose and mounted in Optimal Cutting Temperature (OCT) compound. Next, 10-μm cryosections were blocked (PBS containing 0.1% Triton X-100 (PBX), 1% BSA and 2% donkey serum) for 1 h at room temperature, and then primary antibodies diluted in blocking buffer were incubated overnight. After three 10-min washes in PBX, secondary antibodies diluted in blocking buffer were incubated for 1 h at room temperature. Slides were then washed three times for 10 min in PBX before mounting with VECTASHIELD Antifade Mounting Medium.

For immunofluorescence staining of vibratome sections, fixed embryos were mounted in 6% low-melting-temperature agarose. Then, 150–200-μm vibratome sections were cut using a Leica VT1000S or a Leica VT1200S. Tissue slices were permeabilized in 0.5% PBX and then incubated in either PermBlock solution (PBS containing 3% BSA and 0.1% Tween 20) or blocking buffer (PBS containing 0.5% PBX, 0.5% Tween 20, 1% BSA and 3% donkey serum) for 2 h at room temperature and primary antibodies overnight at 4 °C. Subsequently, tissues were washed three times for 20 min in PBX and incubated with Alexa Fluor–conjugated secondary antibodies overnight at 4 °C. Tissues were then washed in PBX before mounting.

For whole-mount staining of fixed embryos, samples were permeabilized in 0.5% PBX for 12 h at room temperature, blocked in PermBlock solution for 1–2 d at room temperature and stained with primary antibodies at 37 °C for at least 2 d. After three washing steps with PBST (1× PBS containing 0.1% Tween 20), tissues were incubated with Alexa Fluor–conjugated secondary antibodies for at least 1 d at 37 °C and then washed three times with PBST. Stained embryos were stored in PBST at 4 °C until further processing for optical clearing.

For whole-mount staining of the embryonic skin, tissues were fixed in 4% PFA for 2 h at room temperature, washed in 1× PBS and then incubated in blocking buffer (1× PBS containing 0.3% PBX, 1% BSA and 3% donkey serum) for 2 h at room temperature. Tissues were then incubated with primary antibodies diluted in blocking solution overnight at 4 °C. After primary antibody incubation, tissues were washed five times for 10 min in PBX and then incubated with Alexa Fluor–conjugated secondary antibodies for 3 h at room temperature. Tissues were then washed five times for 10 min in PBX and mounted in VECTASHIELD.

For whole-mount imaging of embryonic hearts, dissected hearts were fixed in 4% PFA overnight at 4 °C, washed in 1× PBS and then blocked (1× PBS containing 0.5% PBX, 0.5% PBST, 1% BSA and 3% donkey serum) overnight at 4 °C. Samples were incubated overnight at 4 °C with primary antibodies diluted in incubation buffer (1× PBS containing 0.25% PBX, 0.25% PBST, 0.5% BSA and 1.5% donkey serum). After primary antibody incubation, tissues were washed five times for 30 min in PBX and then incubated with Alexa Fluor–conjugated secondary antibodies overnight at 4 °C. Tissues were then washed five times for 30 min in PBX and mounted in 0.5% low-melting-temperature agarose for imaging.

### Confocal laser scanning microscopy

Imaging of immunostained tissues was performed using Zeiss LSM780, LSM880 or LSM980 confocal microscopes, equipped with the following objectives: ×10 Plan-Apo, numerical aperture (NA) = 0.45; ×20 Plan-Apo, NA = 0.8; ×40 C-Apo water, NA = 1.2; ×63 Plan-Apo oil, NA = 1.4. Datasets were recorded and processed with ZEN Pro (Zeiss). All confocal images represent maximum intensity projections of z-stacks of either single tile or multiple tile scans. Mosaic tile scans with 10% overlap between neighboring z-stacks were stitched in ZEN software. Confocal single and multiple tile scans were processed in Fiji. If necessary, adjustments to brightness, contrast and intensity were made uniformly across individual channels and datasets.

### Embryo dissociation for flow cytometry and FACS

All dissections were performed in PBS with 2% heat-inactivated FBS. For scRNA-seq experiments, embryos were dissected at the level of the otic vesicle and first pharyngeal arch to remove cranial tissues, with embryos from the same stage pooled as follows: E9.5: 18 embryos from four litters; E10.5: 10 embryos from three litters; E11.5: nine embryos from two litters. For scMultiome-seq experiments, 25 E9.5 *Pax3*^*Cre/+*^*;Rosa26*^*tdTomato*^ embryos were dissected at the level of the otic vesicle and first pharyngeal arch to remove cranial tissues. Single-cell suspensions were obtained and blocked as described above. For each stage, embryos were incubated in PBS with 10% FBS, 2 mg ml^−1^ Collagenase IV (Gibco, 9001-12-1) and 0.2 mg ml^−1^ DNase I (Roche, 10104159001) for 20–45 min at 37 °C until fully dissociated. The cell suspension was resuspended every 5 min. After digestion, PBS containing 0.5% FBS and 2 mM EDTA was added in a 1:1 ratio, with the resulting suspension passed through a 40-μm filter and centrifuged at 500*g* for 5 min at 4 °C. Cell pellets were resuspended in Cell Staining Buffer (BioLegend, 420201). Cell counting was performed using a Countess 3 Automated Cell Counter (Thermo Fisher Scientific).

### Flow cytometry

Single-cell suspensions, generated as above, were incubated with Zombie Aqua Fixable Viability Kit (BioLegend, 423101, 1:1,000) for 15 min at room temperature. Cells were washed with Cell Staining Buffer (BioLegend, 420201) and then washed, centrifuged at 500*g* for 5 min and blocked with Fc block CD16/32 (BioLegend, 101302, 1:100) for 5 min on ice and stained with PECAM1-BV605 (BioLegend, 102427, 1:1,000), CD45-FITC (BioLegend, 157607, 1:200), CD41-BV421 (BioLegend, 133911, 1:200), PDPN-eF660 (eBioscience, 50-5381-82, 1:100) and LYVE1-PECy7 (eBioscience, 25-0443-82, 1:400) for 30 min on ice and then washed and resuspended in Cell Staining Buffer. The samples were either analyzed immediately on a BD LSRFortressa X20 cytometer or stored in IC Fixation Buffer (eBioscience, 00-8222-49), washed and analyzed the next day.

### FACS

Single-cell suspensions were obtained as described above and subsequently blocked for 5 min on ice with Fc block CD16/32 (BioLegend, 101302, 1:100), followed by the addition of antibodies for 30 min on ice: VEGFR2-PECy7 (BioLegend, 136414, 1:100), CD45-APC (BioLegend, 103111, 1:200) and CD41–Alexa 647 (BioLegend, 133933, 1:200). For E10.5 and E11.5 suspensions, PECAM1-PECy7 (BioLegend, 102418, 1:100) was also included to capture as broad a pool of phenotypically diverse ECs as possible. The cell suspension was washed using Cell Staining Buffer (BioLegend, 420201) and then incubated with SYTOX Blue Dead Cell Stain (Invitrogen, S34857, 1:10,000) 10 min on ice and then washed. Single cells were sorted using a BD FACSAria III and collected into PBS containing 0.5% BSA for scRNA-seq experiments and PBS containing 0.04% BSA for scMultiome-seq experiments (Miltenyi Biotec, 130-091-376).

### scRNA-seq

#### Library generation and pre-processing

scRNA-seq was performed using the 10x Genomics Chromium platform, and libraries were generated using the Next GEM Single Cell 3′ GEM, Library & Gel Bead Kit version 3.1 (10x Genomics, PN-1000128). Libraries were sequenced with the standalone mode set to the manufacturerʼs protocol on the Illumina NextSeq 500 platform using the NextSeq 500/550 High Output Kit version 2.5, 150 cycles (Illumina, 20024907), to a depth of approximately 50,000 reads per cell. Raw base call files were demultiplexed using bcl2fastq version 2.20 software (Illumina) according to 10x Genomics instructions. Reads were aligned to the mm10 genome with the tdTomato-WPRE sequence added, and cells were called using CellRanger 5.0 (10x Genomics).

#### Quality control and normalization

The filtered barcode matrices were loaded into RStudio version 1.4 and further analyzed using Seurat 4.0 (refs. ^68,69^). Cells with fewer than 2,500 detected genes and more than 100,000 unique molecular identifiers (UMIs) and 7% of mitochondrial reads were removed. The data were normalized using the NormalizeData function, and the highly variable features were calculated using FindVariableFeatures. The data were further scaled using ScaleData, and principal component analysis (PCA) was performed using the variable features previously calculated. Cell cycle stage was predicted using G2M and S-phase genes^70^.

#### Batch correction

To remove technical batch effects due to the different collection and processing times between the samples, the Seurat wrapper FastMNN, a faster version of MNN^71^, was used. The RunFastMNN function was used with auto.merge = T, and *k* was decreased to 10 to avoid overcorrection.

#### Clustering

A nearest neighbor graph was calculated using FindNeighbors using 10 corrected embeddings from FastMNN and 10 neighbors (k.param = 10). The clusters were found using FindClusters with resolution = 2. The clusters were manually annotated according to marker genes determined with FindMarkers. The clusters clearly separated based on cell cycle stage alone were manually merged back together.

#### Visualization and trajectory analyses

The integrated Seurat object was converted to H5AD format for import into Python (Jupyter Notebook interface^72^) using the packages SeuratDisk and SeuratData. The Scanpy 1.8 package^73^ was used to run the PAGA^74^ algorithm (scanpy.tl.paga) to produce an embedding that captures the connectivity of the data accurately using the corrected principal components (PCs) from FastMNN and the clusters previously calculated. To visualize the data, a force-directed graph^75^ was calculated using scanpy.tl. draw_graph and pre-computed coordinates from PAGA.

#### Waddington-OT analyses

Trajectories from E9.5 to E11.5 were also assessed using Waddington-OT^76^, a pipeline based on optimal transport. The results corroborate independent analyses with PAGA (Extended Data Fig. 5e). We used Waddington-OT with default parameters (entropic regularization $\epsilon =0.05$, early timepoint balance regularization *λ*_1_ = 1 and late timepoint balance regularization *λ*_2_ = 50). Given a series of population snapshots at times ${t}_{1},\ldots ,{t}_{T}$, Waddington-OT fits a coupling between consecutive pairs of populations. Each coupling, mathematically a joint distribution on the product space of early and late gene expression, connects cells at the earlier timepoint to their predicted descendants and cells at the later timepoint to their predicted ancestors. To compute the couplings, Waddington-OT solves an entropically regularized unbalanced optimal transport problem, minimizing the difference in gene expression between predicted ancestors and descendants subject to the constraint that the population at *t*_*i*_ maps to the population at *t*_*i*+1_. Entropic regularization allows for stochasticity in cell fate decisions, whereas unbalanced transport accounts for uncertainty in the relative growth and death rates of different cells. In the absence of clear prior information about the relative growth rates of cells, we left the initial growth rate estimates uniform. By concatenating the coupling from E9.5 to E10.5 with the coupling from E10.5 to E11.5, we are able to predict ancestors and descendants across the timecourse.

### scMultiome-seq

#### Nuclei isolation and transposition

FACS-sorted cells were gently resuspended in 50 μl of chilled lysis buffer (10 mM Tris-HCl (pH 7.4), 10 mM NaCl, 3 mM MgCl_2_, 0.1% Tween 20, 0.1% NP-40 substitute, 0.01% digitonin, 1% BSA, 1 mM DTT and 1 U µl^−1^ RNase inhibitor) for 3 min on ice. Nuclei were washed with diluted nuclei buffer, visualized with trypan blue staining under a light microscope for quality control and transposed according to the manufacturer’s instructions (Chromium Next GEM Single-Cell Multiome ATAC + Gene Expression User Guide, Rev F).

#### Library generation and pre-processing

Multiome sequencing of transposed nuclei was performed using the 10x Genomics Chromium platform, and sequencing libraries were generated using the Chromium Next GEM Single-Cell Multiome ATAC + Gene Expression Kit (10x Genomics, PN-1000285). Libraries were sequenced with the standalone mode set to the manufacturerʼs protocol on the Illumina NextSeq 500 platform using the NextSeq 500/550 High Output Kit version 2.5, 150 cycles (Illumina, 20024907), to a depth of approximately 27,300 reads per cell for the gene expression library and approximately 33,500 reads per cell for the ATAC library. FASTQ files were processed using the 10x CellRanger Arc pipeline (version 2.0.0) count function with default settings and mapped to the refdata-cellranger-arc-mm10-2020-A-2.0.0 reference.

#### Quality control and normalization

CellRanger Arc outputs were loaded into RStudio version 1.4 and further analyzed using Seurat version 5.0.1 (ref. ^77^) for gene expression and ATAC analyses and Signac version 1.12.0 (ref. ^78^) for ATAC analyses. Before filtering, the ATAC data were processed to retain only peaks in standard chromosomes. Nuclei with fewer than 1,000 or more than 20,000 cDNA reads, fewer than 1,000 ATAC fragments or more than 15% mitochondrial reads were removed to retain 3,801 high-quality nuclei for analysis. RNA data were normalized using the NormalizeData function before calculation of variable features with FindVariableFeatures and scaling data with ScaleData. RunPCA was used to calculate the first 50 PCs. ATAC data were normalized with RunTFIDF, and top features were identified with FindTopFeatures before calculating dimensiontality reduction with RunSVD.

#### Clustering

A weighted nearest neighbor (WNN) graph was constructed with FindMultimodalNeighbors using the 1–25 and 2–50 dimensions from the PCA and latent semantic indexing (LSI) reductions, respectively. This graph was subsequently used to calculate a uniform manifold approximation and projection (UMAP) for visualization and clustering with FindClusters (resolution = 0.8). Clusters were assigned biological annotation according to the top 10 enriched genes (FindAllMarkers) and visualization of known marker genes.

#### GRN analysis

To construct GRNs during LEC specification and differentiation, we applied the SCENIC+ pipeline^79^ to our scMultiome dataset. High-quality singlets identified from the Seurat analysis described above were selected for SCENIC+ analysis.

#### Identification of enhancer candidates and topic modeling

pycisTopic^79^ was used to identify enhancer candidates. ATAC fragment files were processed to generate pseudobulk BigWig and BAM files for each cell type and used to call peaks with MACS2 (ref. ^80^) and merge into consensus peaks with get_consensus_peaks(). Together with filtered fragments, these consensus regions were used to create a cisTopic object. We ran latent Dirichlet allocation (LDA) models on the cisTopic object with between two and 50 topics using run_cgs_models_mallet() and used evaluatemodels() to select 30 as the optimal number of co-accessible region sets. Topics were manually inspected for their enrichment in cell types to ensure that a range of cell-type-specific topics had been identified. Finally, differentially accessible regions between cell types were identified using pycisTopic’s find_diff_features().

#### Generating a custom cisTarget database

pycisTarget^80^ was used to create pre-computed enrichment scores for all SCENIC+ motifs across our consensus peaks. FASTA files were generated from consensus regions identified above with an additional kilobase of padding as a background sequence for cluster-buster. Finally, we used pycisTarget’s create_cistarget_motif_databases.py to generate our custom database.

#### Inferring eGRN

Using the outputs from pycisTopic and pycisTarget, in combination with snRNA-seq data exported from Seurat to Scanpy, we ran the SCENIC+ snakemake pipeline using default settings to predict the enhancer-driven regulons constituting the GRN. The direct eRegulon metadata were used to examine the activity of gene-based and region-based eRegulons (AUCell scores) in heatmaps using heatmap_dotplot(). To export eRegulon network graphs, the mudata object output from the snakemake pipeline was converted to a scenicplus object for backwards compatibility. For network graphs of known EC differentiation (Extended Data Fig. 6h), the top 350 most variable genes and regions were selected as nodes. Conversely, when investigating putatitive regulators (Extended Data Fig. 6i), the genes and regions with the highest triplet score were selected.

#### In silico prediction of TF binding

To predict the binding of TFs in silico on a per-cell-type basis, we first generated ATAC pseudobulks using sinto (https://github.com/timoast/sinto). Peaks were called for each pseudobulk using Genrich (https://github.com/jsh58/Genrich). The resulting .bam and .bed files were used to run the TOBIAS footprinting pipeline^81^. First, pseudobulks were corrected for Tn5 cutting bias with ATACorrect, followed by calculation of footprinting scores with FootprintingScores. Scores were calculated for all motifs in JASPAR 2024 core motif collection with motifs of interest manually inspected in Integrative Genomics Viewer (IGV).

#### Analysis of publicly available ETV2 ChIP-seq data

Publicly available ETV2 ChIP-seq data were retrieved from GSE59402 (ref. ^37^). BigWig files were converted from the mm9 to the mm10 genome for display with our snATAC-deq data using CrossMap^82^ and the mm9 to mm10 chain file. Converted BigWig files were displayed using IGV.

### Bulk ATAC-seq analysis

#### Nuclei isolation and transposition

Bulk ATAC-seq libraries were generated following the Omni-ATAC protocol^83^. In brief, BECs (tdTomato^−^CD41^−^CD45^−^PECAM1^+^PDPN^−^) and LECs (tdTomato^+^CD41^−^CD45^−^PECAM1^+^PDPN^+^) were FACS-sorted from E13.5 *Pax3*^*Cre/+*^*; Rosa26*^*tdTomato*^ embryos. In total, 10,000 cells were gently resuspended in 50 μl of chilled lysis buffer (10 mM Tris-HCl (pH 7.4), 10 mM NaCl, 3 mM MgCl_2_, 0.1% Tween 20, 0.1% NP-40 substitute, 0.01% digitonin, 1% BSA, 1 mM DTT and 1 U µl^−1^ RNase inhibitor) for 3 min on ice. Nuclei were washed with 990 μl of chilled resuspension buffer (10 mM Tris-HCl (pH 7.4), 10 mM NaCl, 3 mM MgCl_2_ and 0.1% Tween 20), incubated in transposition buffer (1× TD buffer, 100 nM Tn5 Transposase (FC-121-1030, Illumina), 0.01% digitonin, 0.1% Tween 20 and 0.33× PBS) for 30 min at 37 °C and 1,000 rpm. Reactions were subsequently cleaned up with the MinElute Reaction Cleanup Kit (Qiagen, 28204).

#### Library generation and pre-processing

Sequencing libraries were generated following standard protocols^84^ and amplified with a target concentration of 4 nM. Tagmentation efficiency was assessed with an Agilent TapeStation, and libraries were sequenced using paired-end 40-bp reads on an Illumina NextSeq 500. Reads were mapped to the mm10 genome assembly using Bowtie 2, and duplicates were removed using Picard’s MarkDuplicates. To visualize peaks at regions of interest, BigWig files were generated using bamCoverage with a binSize of 10 for loading into IGV.

### In situ HCR

Whole-mount in situ HCR is commercially available from Molecular Technologies^85^. The HCR version 2.0 protocol for whole-mount mouse embryos was performed according to the manufacturer’s instructions. The following probes were used: Etv2-B4 (NM_007959.2), Prox1-B2 (NM_008937.3), Pecam1-B3 (NM_008816,3), Lbx1-B1 (NC_000085.7), C1ql2-B3 (NM_207233.1), Tll1-B1 (NM_009390.3), Kdr-B3 (NM_010612.3), Flt4-B5 (NM_008029.3), Vegfa-B2 (NM_009505) and Vegfc-B1 (NM_009506.2). Embryos were mounted in SlowFade Gold Antifade Mountant (Invitrogen, S36936) and analyzed using a Zeiss inverted LSM 880 or LSM 980 or an Olympus FV1000 laser scanning confocal microscope.

### RNAscope fluorescence in situ hybridization

RNA in situ hybridization on embryonic tissue was performed using the commercially available RNAscope Multiplex Fluorescent version 2 assays (Advanced Cell Diagnostics (ACD), 323100). Frozen tissue sections of 20-µm thickness were processed according to the manufacturer’s protocols for fresh-frozen samples. For thick vibratome sections of 300 µm, a modified protocol was applied. In brief, tissue sections were dehydrated in 50%, 75% and 100% methanol for 10 min and then washed with PBS-T (PBS with 0.1% Tween 20). Sections were then pre-treated with hydrogen peroxide for 10 min at room temperature and Protease III reagent for 20 min at room temperature, followed by washing with PBS supplemented with protease inhibitor (Sigma-Aldrich, 11873580001). Sections were post fixed using 4% PFA for 30 min at room temperature and washed with 0.2× saline sodium citrate (SSC) buffer. For RNA detection, sections were incubated with the following probes for 3 h at 40 °C: Mm-Prox1-C2 (ACD, 488591), Mm-Pecam1-C1 (ACD, 316721), Mm-Cdh5-C3 (ACD, 31253), Mm-Lyve1-C1(ACD, 428451), Mm-Vegfc-C2 (ACD, 492701-C2) and Mm-Ccbe1-C1 (ACD, 485651). Subsequent amplification steps were performed at 40 °C (AMP1-FL and AMP2-FL: 50 min each; AMP3-FL: 20 min), and each amplifier was removed by washing using 0.2× SSC buffer. For signal detection, sections were incubated with the channel-specific HRP for 20 min at 40 °C and incubated with the respective fluorophores (PerkinElmer: Fluorescein, 1:500; Cy3, 1:1,000; Cy5, 1:1,500; Opal 520, 1:750; Opal 620, 1:750) for 40 min at 40 °C, followed HRP blocker incubation for 20 min at 40 °C.

### Analysis of cell cycle kinetics in mid-gestation embryos

The mean total cell cycle (*T*_C_) and S-phase duration (*T*_S_) of initial LECs at E10.5 were determined using an adapted dual-pulse labeling protocol^86,87^. The experimental setup and calculation of cell cycle kinetics are outlined in Fig. 5f and Extended Data Fig. 9d, respectively. EdU (50 mg kg^−1^, intraperitoneal) was administered to pregnant females three times, with a time interval of 2 h, to maintain bioavailability during the experiment. After 6 h, BrdU (50 mg kg^−1^, intraperitoneal) was administered, and, 2 h later, embryos were dissected and fixed overnight in 4% PFA at 4 °C. Vibratome sections (150 µm) were permeabilized in 0.5% Triton X-100 in PBS for 30 min at room temperature. Heat-induced antigen retrieval (HIER) was used to facilitate BrdU antibody labeling. Sections were submerged in sodium citrate buffer (10 mM sodium citrate, 0.05% Tween 20, pH 6.0) and heated to 98 °C for 30 min. Sections were cooled to room temperature with fresh sodium citrate buffer and washed for 5 min with PBS. After HIER, sections were incubated in blocking solution (3% BSA, 0.1% Tween 20 in PBS) for 2 h at room temperature and then incubated with antibodies to BrdU (Abcam, ab6326, 1:100), EMCN (D. Vestweber, VE44, 1:100) and PROX1 (Reliatech, 102-PA32, 1:100). Subsequently, slides were washed in PBS-T (PBS with 0.1% Tween 20) 3 times for 10 min each and incubated with Alexa Fluor–conjugated secondary antibodies for 2 h at room temperature or overnight at 4 °C. For EdU detection, the Click-iT Alexa Fluor 555 reaction cocktail (Thermo Fisher Scientific) was freshly prepared and incubated for 30 min at room temperature. Sections were rinsed in PBS and then mounted with Mowiol. Sections were imaged on a Zeiss LSM 880 confocal microscope using a ×40 water immersion objective (NA = 1.2). Cell counts were performed on at least three 150-μm sections, and individual data points were calculated as the mean of all sections analyzed per embryo. LEC progenitors at E10.5 are represented by PROX1^+^ nuclei (hereafter, P_cells_). EdU^+^ and BrdU^+^ cells were scored as any nuclei showing immunoreactivity for these markers regardless of staining pattern.

The *T*_S_ of *P*_cells_ is given by equation (1):

1 $${{T}}_{\text{S}}=\frac{{{{T}}}_{{{i}}}}{{{{L}}}_{{\rm{cells}}}/{{{S}}}_{{\rm{cells}}}}$$

*T*_i_ is the injection interval during which cells can incorporate at least one of the thymidine analogues. EdU^+^/BrdU^+^ double-positive cells reflect all cells in the S-phase at the end of the experiment (*S*_cells_). Cells of the initial EdU-labeled S-phase population leave the S-phase during T_i_, with this fraction labeled with EdU but not BrdU (*L*_cells_).

The T_C_ of P_cells_ cells is given by equation (2):

2 $${T}_{\text{C}}=\frac{{T}_{\text{S}}}{{{S}}_{{\rm{cells}}}/\left({{P}}_{{\rm{cells}}}\times {\rm{GF}}\right)}$$

To avoid overestimation of T_c_, which could occur if not all cells are actively progressing through the cell cycle, a growth fraction (GF) that represents the proportion of P_cells_ that are in the cell cycle was used for calculation of *T*_c_. GF can be determined by examining the proportion of P_cells_ labeled with the cell cycle marker KI67 using equation (3):

3 $$\text{GF}=\frac{{{P}}_{{\rm{cells}}}{{\rm{KI}}67}^{+}}{{{P}}_{{\rm{cells}}}}$$

### Optical tissue clearing

The methods used for optical tissue clearing were described previously^88^. In brief, before tissue clearing, whole-mount stained samples were embedded in 1% low-melting agarose to facilitate sample handling. Samples were dehydrated in increasing concentrations of methanol (50%, 70%, >99.5% and >99.5% (v/v)) for at least 1 h each. After dehydration, tissues were incubated in a 1:1 mixture of >99.5% methanol and BABB (1:2 benzyl alcohol:benzyl benzoate) for 1 h and, finally, in BABB for at least 4 h.

### Light sheet microscopy

Optically cleared samples were imaged using an UltraMicroscope II Super Plan (Miltenyi Biotec) equipped with ×4 MI Plan Objective (NA = 0.35, working distance (WD) ≥ 15 mm). An NKT SuperK supercontinuum white light laser served as excitation light source. For excitation and emission detection of specific fluorophores, custom band-pass filters (excitation 470/40, 577/25 or 640/30 nm; emission 525/50, 632/60 or 690/50 nm) were used in combination with a pco.edge 4.2 sCMOS camera. Images were acquired with 2-µm steps in the *z* axis.

### Quantification of cell numbers

Confocal and light sheet image stacks were rendered into three-dimensional (3D) volumes and analyzed using Imaris version 9.5 (Bitplane; RRID: SCR_007370). Quantification of absolute cell numbers is based on staining of specific TFs to visualize the nuclei of cells of interest. Thus, the number of nuclei reflect the number of cells. Nuclei were automatically annotated using the ‘Spots’ function, which automatically detects point-like structures with a pre-defined diameter. Accurate quantification required an appropriate estimate of cell nuclei diameter and filtration of selected nuclei by tuning the quality parameters. The accuracy of this automatic counting procedure was verified by visual inspection, which herein served as ground truth. Using the ‘manual Surface creation’ function, vascular structures were segmented based on specific EC marker expression. Thereby, cell populations inside and outside of segmented vascular structures were defined by filtering the shortest distance between ‘Spots’ and ‘Surface’.

### Directional migration of LEC progenitors

To assess directional migration of PROX1^+^ LEC progenitors, we established an image analysis pipeline to automatically define and quantify cell polarity in large tissue sections. Vibratome sections (200 µm) of E10.5 embryos were stained for Golgi (GM130) and LEC nuclei (PROX1), and sections were imaged on a Zeiss LSM 880 confocal microscope using a ×40 water immersion objective (NA = 1.2). In a first post-processing step, Golgi and nuclei in each image stack were segmented using the ‘Surface’ function in Imaris version 9.5 (Bitplane; RRID: SCR_007370). Cell populations inside and outside of defined vascular structures were defined as described above (‘Quantification of cell numbers’ subsection). Surface masks were exported and processed in Fiji version 1.53. Subsequently, nearest neighbor analyses were used to pair individual nuclei with their corresponding Golgi (closest border–border distance), and the centroid of each object was computed using 3D ImageJ Suite version 4.0.36 (ref. ^89^). Two-dimensional vectorization images were obtained by drawing arrows from the nuclei centroid toward the Golgi centroid using Fiji. Nuclei–Golgi pairs with a border-to-border distance larger than 5 µm were excluded from further analysis. Centroid vectors were produced using the *x–y* coordinates of nuclei and Golgi centroids and transformed to unit vectors (A). The dorsal body axis served as reference vector (B). The angle was obtained by calculating the inverse cosine of the dot product of centroid unit vectors (A) and reference vector (B) ($\theta =\arccos (A{\rm{\cdot }}B)$). All calculations were performed using Python version 3.8. Angles were transformed to represent the body axes (0°, dorsal; 90°, lateral; 180°, ventral; 270°, medial), and a histogram on a polar axis was used to display the angular distribution of individual LECs representing their migration direction.

### Statistical analysis

Statistical analyses were performed by unpaired, two-tailed Student’s *t*-test or non-parametric one-way ANOVA followed by Tukey’s honestly significant difference (HSD) using GraphPad Prism software. Data are presented as mean ± s.d. (error bars). *P* < 0.05 was considered statistically significant (*P < 0.05, **P < 0.01, ***P < 0.001, ****P < 0.001).

### Reporting summary

Further information on research design is available in the Nature Portfolio Reporting Summary linked to this article.

## Supplementary information

Reporting Summary

## Source data

Source Data Figs. 4 and 5 and Source Data Extended Data Figs. 7–9 Statistical source data.

## Data Availability

scRNA-seq (GSE276281) and scMultiome-seq and bulk ATAC-seq (GSE276282) data were deposited in the Gene Expression Omnibus (GEO). ETV2 ChIP-seq data were downloaded from the GEO (GSE59402). Correspondence and requests for materials should be addressed to F.K. and O.A.S.

## Code availability

All scripts required to reproduce the data presented are available on GitHub at https://github.com/StoneLabGH/LEC_Specification.
