# Supplementary material for: Direct specification of lymphatic endothelium from mesenchymal progenitors
Source: Nat Cardiovasc Res. 2025 Jan 2;4(1):45–63. doi: 10.1038/s44161-024-00570-5 (PMC11738995; doi:10.1038/s44161-024-00570-5)
Supplement: Supplementary file 1 — Reporting Summary [file 44161_2024_570_MOESM1_ESM.pdf]

nature portfolio

Corresponding author(s): Oliver Stone and Friedemann Kiefer

Last updated by author(s): Oct 21, 2024

Reporting Summary

Nature Portfolio wishes to improve the reproducibility of the work that we publish. This form provides structure for consistency and transparency in reporting. For further information on Nature Portfolio policies, see our Editorial Policies and the Editorial Policy Checklist.

Statistics

For all statistical analyses, confirm that the following items are present in the figure legend, table legend, main text, or Methods section.

n/a	Confirmed	
☐	☒	The exact sample size (*n*) for each experimental group/condition, given as a discrete number and unit of measurement
☒	☐	A statement on whether measurements were taken from distinct samples or whether the same sample was measured repeatedly
☐	☒	The statistical test(s) used AND whether they are one- or two-sided<br>*Only common tests should be described solely by name; describe more complex techniques in the Methods section.*
☒	☐	A description of all covariates tested
☐	☒	A description of any assumptions or corrections, such as tests of normality and adjustment for multiple comparisons
☐	☒	A full description of the statistical parameters including central tendency (e.g. means) or other basic estimates (e.g. regression coefficient) AND variation (e.g. standard deviation) or associated estimates of uncertainty (e.g. confidence intervals)
☒	☐	For null hypothesis testing, the test statistic (e.g. *F*, *t*, *r*) with confidence intervals, effect sizes, degrees of freedom and *P* value noted<br>*Give P values as exact values whenever suitable.*
☒	☐	For Bayesian analysis, information on the choice of priors and Markov chain Monte Carlo settings
☒	☐	For hierarchical and complex designs, identification of the appropriate level for tests and full reporting of outcomes
☒	☐	Estimates of effect sizes (e.g. Cohen's *d*, Pearson's *r*), indicating how they were calculated

Our web collection on statistics for biologists contains articles on many of the points above.

Software and code

Policy information about availability of computer code

Data collection	Confocal laser scanning microscopy:<br>Imaging of immunostained tissues was performed using Zeiss LSM780, LSM880 or LSM980 confocal microscopes. Datasets were recorded and processed with ZEN Pro (Carl Zeiss). All confocal images represent maximum intensity projections of z-stacks of either single tile or multiple tile scans. Mosaic tile-scans with 10% overlap between neighbouring z-stacks were stitched in ZEN software. Confocal single and multi-tile-scans were processed in Fiji. If necessary, adjustments to brightness, contrast and intensity were made uniformly across individual channels and datasets.<br><br>Directional migration of LEC progenitors:<br>To assess directional migration of PROX1+ LEC progenitors, we established an image analysis pipeline to automatically define and quantify cell polarity in large tissue sections. Vibratome sections were imaged on a Zeiss LSM 880 confocal microscope and in a first post-processing step, Golgi and nuclei in each image stack were segmented using the “Surface” function in Imaris v9.5 (Bitplane; RRID:SCR_007370).<br><br>Flow Cytometry and FACS: Flow cytometry was performed on a BD LSRFortressa X20 cytometer and FACS on a BD Aria III. Datasets were recorded and processed using BD FACS Diva software v8.0.
Data analysis	Single cell genomics data analysis:<br>All scripts required to reproduce the data presented are available on GitHub at https://github.com/StoneLabGH/LEC_Specification.<br><br>Data analysis softwares:<br>GraphPad Prism 10 was used for statistical analyses and presenting data. FlowJo v9 was used to analysis flow cytometry data. Confocal images were processed in Fiji v2.1.0 and Imaris v9.5 (Bitplane). The following softwares were used for single cell RNA sequencing analyses: Cell

Ranger 5.0 (10x Genomics), RStudio v1.4, Seurat 4.0, Python v3.11 (Jupyter Notebook interface), Scanpy 1.8, scanpy.tl.paga. The following softwares were used for single cell multiome analyses: 10x Cell Ranger Arc pipeline (v2.0.0), RStudio v2022.02.3, R v4.2.0, Seurat v5.0.1, Signac v1.9.0, SCENIC+ v1.0a1, pycisTopic v2.0a0, MACS2 (v.2.2.9.1), IGV(v2.13.1), TOBIAS (v.0.17.0), sinto (https://github.com/timoast/sinto), Genrich (https://github.com/jsh58/Genrich). For bulk ATAC-seq analyses, the following softwares were used: bowtie2 (v.2.4.4), bamCoverage (v2.1.0)

Quantification of cell numbers:
Confocal and light sheet image stacks were rendered into 3D volumes and analysed using Imaris v9.5 (Bitplane; RRID:SCR_007370). Quantification of absolute cell numbers is based on staining of specific transcription factors to visualize the nuclei of cells of interest. Thus, the number of nuclei reflect the number of cells. Nuclei were automatically annotated using the “Spots” function, which automatically detects point-like structures with a predefined diameter. Accurate quantification required an appropriate estimate of cell nuclei diameter and filtration of selected nuclei by tuning the quality parameters. The accuracy of this automatic counting procedure was verified by visual inspection, which herein severed as ground truth. Using the “manual Surface creation” function, vascular structures were segmented based on specific EC marker expression. Thereby cell populations inside and outside of segmented vascular structures were defined by filtering the shortest distance between “Spots” and “Surface”.

Directional migration of LEC progenitors:
Cell populations inside and outside of defined vascular structures were defined as described above (Quantification of cell numbers). Surface masks were exported and processed in Fiji v1.53. Subsequently, nearest neighbour analyses were used to pair individual nuclei with their corresponding Golgi (closest border – border distance), and the centroid of each object was computed using the 3D ImageJ Suite v4.0.36 74. 2D vectorization images were obtained by drawing arrows from the nuclei centroid towards the Golgi centroid using Fiji. Nuclei – Golgi pairs with a border-to-border distance larger than 5 µm were excluded from further analysis. Centroid vectors were produced using the XY-coordinates of nuclei and Golgi centroids and transformed to unit vectors (A). The dorsal body axis served as reference vector (B). The angle was obtained by calculating the inverse cosine of the dot product of centroid unit vectors (A) and reference vector (B) (θ=arccos(A ·B)) . All calculations were performed using python v3.8. Angles were transformed to represent the body axes (0°, dorsal; 90° lateral; 180°, ventral; 270°, medial) and a histogram on a polar axis was used to display the angular distribution of individual LECs representing their migration direction.

For manuscripts utilizing custom algorithms or software that are central to the research but not yet described in published literature, software must be made available to editors and reviewers. We strongly encourage code deposition in a community repository (e.g. GitHub). See the Nature Portfolio guidelines for submitting code & software for further information.

Data

Policy information about availability of data

All manuscripts must include a data availability statement. This statement should provide the following information, where applicable:

- Accession codes, unique identifiers, or web links for publicly available datasets
- A description of any restrictions on data availability
- For clinical datasets or third party data, please ensure that the statement adheres to our policy

Data availability
scRNA-seq (GSE276281) and scMultiome-seq (GSE276282) data were deposited in the Gene Expression Omnibus (GEO). Correspondence and requests for materials should be addressed to F.K. and O.A.S.

Research involving human participants, their data, or biological material

Policy information about studies with human participants or human data. See also policy information about sex, gender (identity/presentation), and sexual orientation and race, ethnicity and racism.

Reporting on sex and gender	n/a
Reporting on race, ethnicity, or other socially relevant groupings	n/a
Population characteristics	n/a
Recruitment	n/a
Ethics oversight	n/a

Note that full information on the approval of the study protocol must also be provided in the manuscript.

Field-specific reporting

Please select the one below that is the best fit for your research. If you are not sure, read the appropriate sections before making your selection.

☒ Life sciences ☐ Behavioural & social sciences ☐ Ecological, evolutionary & environmental sciences

For a reference copy of the document with all sections, see nature.com/documents/nr-reporting-summary-flat.pdf

Life sciences study design

All studies must disclose on these points even when the disclosure is negative.

Sample size	We used the NC3Rs experimental design assistant (EDA) to form an experimental plan that integrates statistics-based power calculations informed by our knowledge of estimated variability.
Data exclusions	No data were excluded
Replication	Experiments were performed following careful staging of embryos, and all presented analyses used the offspring of multiple pregnant dams. Analyses were reproduced through multiple independent experiments to confirm the obtained results. Specific numbers of replication for each experiments are included in the figures and figure legends.
Randomization	Due to the nature of most of the experiments performed here, allocation of embryos to distinct treatment groups is randomized. For example, Vegfc +/+ and Vegfc -/- embryos were identified from a pool of littermates though genotyping following imaging analyses.
Blinding	For imaging and flow cytometry analyses, investigators were blinded to genotype and treatments until completion of data. collection/ quantification.

Reporting for specific materials, systems and methods

We require information from authors about some types of materials, experimental systems and methods used in many studies. Here, indicate whether each material, system or method listed is relevant to your study. If you are not sure if a list item applies to your research, read the appropriate section before selecting a response.

Materials & experimental systems

n/a	Involved in the study	
☐	☒	Antibodies
☒	☐	Eukaryotic cell lines
☒	☐	Palaeontology and archaeology
☐	☒	Animals and other organisms
☒	☐	Clinical data
☒	☐	Dual use research of concern
☒	☐	Plants

Methods

n/a	Involved in the study	
☒	☐	ChIP-seq
☐	☒	Flow cytometry
☒	☐	MRI-based neuroimaging

Antibodies

Antibodies used

The following antibodies were used for immunofluorescence staining of cryosections, vibratome sections and/or whole mount tissues: ETV2 (Abcam, #ab181847, 1:100), VEGFR2 (BD Pharmingen, #550549, 1:200), PECAM1 (R&D Systems, #AF3628, 1:250), PECAM1 (D. Vestweber, Clone 5D2.6 and 1G5.1, 15 ?g / mL), PROX1 (R&D Systems, #AF2727, 1:200), PROX1 (Proteintech, #11067-2-AP, 1:100), PROX1 (Reliatech, #102-PA32, 1:100), PROX1 (Abcam, #ab225414, 1:100), EMCN (Santa Cruz Biotechnology, SC-65495, 1:50), EMCN (D. Westweber, #VE44, 1:100), EMCN (Santa Cruz Biotechnology, SC-53941, 1:50), VWF (Abcam, #11713, 1:100), ESR1 (Abcam, #ab16660, 1:100), NRP2 (R&D Systems, #AF567, 1:250), ERG (Abcam, #ab92513, 1:200), GM130 (BD Pharmingen, #610823, 1:100), RFP (Rockland, #600-401-379, 1:500), BrdU (Abcam, #ab6326, 1:100), KI67 (Thermo Fisher, #14-5698-82, 1:100) and GFP (Thermo Fisher, #A-21311, 1:100), Donkey anti-Rat IgG Alexa Fluor Plus 405 (#A48268), Donkey anti-Rabbit IgG Alexa Fluor 488 (#A21206), Donkey anti-Goat IgG Alexa Fluor Plus 488 (#A32814), Donkey anti-Sheep IgG Alexa Fluor 488 (#A11015), Donkey anti-Rabbit IgG Alexa Fluor 555 (#A32814), Donkey anti-Rat IgG Alexa Fluor 555 (#A48270), Donkey anti-Goat IgG Alexa Fluor 647 (#A32849), Donkey anti-Rabbit IgG Alexa Fluor Plus 647 (#A32795), and Donkey anti-Sheep IgG Alexa Fluor 647 (#A21448).

Validation

Antibodies were purchased from commercial vendors and validated by the manufacturer, and/or by previous publications:

- ETV2 (Abcam, #ab181847) : Immunoblot and immunocytochemistry (manufacturer), PMID: 32908310, 29669933, 33522307.
- VEGFR2 (BD Pharmingen, #550549) : detection by immunohistochemistry, immunoblot and immunocytochemistry (manufacturer), PMID: 31943281, 31665628, 31118412.
- PECAM1 (R&D Systems, #AF3628) : detection by immunohistochemistry, immunoblot and immunocytochemistry (manufacturer), PMID: 34259830, 34450028, 37587341.
- PECAM1 (D. Vestweber, Clone 5D2.6) : PMID : 16818677
- PROX1 (R&D Systems, #AF2727) : detection by immunohistochemistry, immunoblot and immunocytochemistry (manufacturer), PMID: 35794479, 25992544, 32251437.
- PROX1 (Proteintech, #11067-2-AP) : detection by immunohistochemistry, immunoblot and immunocytochemistry (manufacturer), PMID: 31341278, 28024299, 34038712
- PROX1 (Reliatech, #102-PA32) : detection by immunoblot and immunofluorescence (manufacturer), PMID:31980640, 38177500, 25061877
- PROX1 (Abcam, #ab199359) : detection by immunohistochemistry, immunoblot and immunocytochemistry (manufacturer), PMID: 37576598, 37699906, 36994549
- EMCN (Santa Cruz Biotechnology, SC-65495) : detection by immunohistochemistry and immunoblot (manufacturer), PMID: 18924607, 24647000, 21909098

- EMCN (D. Westweber, #VE44) PMID: 11554756
- EMCN (Santa Cruz Biotechnology, SC-53941) : detection by immunocytochemistry (manufacturer), PMID: 29773646, 25992544, 31701448.
- VWF (Abcam, #ab11713) : detection by immunocytochemistry (manufacturer), PMID: 37834029, 35325071, 35001873.
- ESR1 (Abcam, #ab16660) : detection by immunocytochemistry (manufacturer), PMID: 33999917, 36729672, 33623049
- NRP2 (R&D Systems, #AF567) : detection by immunoblot, immunohistochemistry, flow cytometry, and immunocytochemistry (manufacturer), PMID: 1130354, 33931446, 30006544
- ERG (Abcam, #ab92513) : detection by immunohistochemistry, flow cytometry, and immunocytochemistry (manufacturer), PMID: 30301887, 30178747, 29400648
- GM130 (BD Pharmingen, #610823) : detection by immunocytochemistry and immunofluorescence (manufacturer), PMID:39198435, 38969763, 38874642.
- RFP (Rockland, #600-401-379) : detection by immunohistochemistry and immunofluorescence (manufacturer), PMID: 38238288, 38579720, 38326622.
- BrdU (Abcam, #ab6326): detection by immunocytochemistry and immunofluorescence (manufacturer), PMID: 38123565, 37874652, 38036565.
- Ki67 (Invitrogen 14-5698-82 rat monoclonal clone SolA15) - detection by immunohistochemistry and immunocytochemistry (manufacturer), PMID: 32750316, 30389919, 27699223
- GFP (Thermo Fisher, #A-21311): detection by immunohistochemistry and immunocytochemistry (manufacturer), PMID: 31797533, 28442548, 28233777

Animals and other research organisms

Policy information about studies involving animals; ARRIVE guidelines recommended for reporting animal research, and Sex and Gender in Research

Laboratory animals

This study was performed using mouse embryos and the following lines were used: Pax3Cre (Pax3tm1(cre)Joe); Pax3CreERT2 (Pax3tm1.1(cre/ERT2)Lepr); Lbx1Cre (Lbx1tm3.1(cre)Cbm); Myf5Cre (Myf5tm3(cre)Sor); Tie2-Cre (Tg(Tek-cre)12Flv/J); Tie2-Cre (Tg(Tek-cre)5326Sato); Prox1fl (Prox1tm1a(EUCOMM)Wtsi); Rosa26tdTomato (Gt(ROSA)26Sortm9(CAG-tdTomato)Hze); Rosa26tdTomato (Gt(ROSA)26Sortm14(CAG-tdTomato)Hze); Rosa26tdRFP (Gt(ROSA)26Sortm1Hjf); Rosa26Fucci2 (Tg(Gt(ROSA)26Sor-Fucci2)Sia), VegfcLacZ (Vegfctm1Ali). With the exception of the VegfcLacZ (Vegfctm1Ali) line, which was maintained on a CD1 background, all lines were maintained on a C57Bl6/J background. Breeding pairs between 2 and 8 months were used to generate animals of the indicated genotypes. Both male and female embryos were used for analyses. Mice were maintained in IVC-cages and ventilated racks at 22°C and 55% humidity with a 12/12 hour light/dark cycle. For embryo collection, mice were paired overnight and females were checked the next morning for the presence of a vaginal plug. For inducible Cre induction, pregnant females were gavaged at the specified time points with 80mg/kg tamoxifen (Sigma, #T5648) dissolved in peanut oil with 10% ethanol at a final concentration of 10mg/ml.

Wild animals

No wild animals were used in this study.

Reporting on sex

As this study involved the analysis of blood and lymphatic vessel development in mouse embryos, we did not record or compare sex dependent differences.

Field-collected samples

No field collected samples were used in this study.

Ethics oversight

All procedures were carried out in accordance with local legislation: University of Oxford Animal Welfare and Ethical Review Boards in accordance with Animals (Scientific Procedures) Act 1986 under Home Office project licences PPL PC013B246 or PP6588077; German animal protection legislation (Tierschutzgesetz und Tierschutz-versuchstierverordnung); Uppsala Animal Experiment Ethics Board (permit number 130/15).

Note that full information on the approval of the study protocol must also be provided in the manuscript.

Plants

Seed stocks

n/a

Novel plant genotypes

n/a

Authentication

n/a

Flow Cytometry

Plots

Confirm that:

☒ The axis labels state the marker and fluorochrome used (e.g. CD4-FITC).

☒ The axis scales are clearly visible. Include numbers along axes only for bottom left plot of group (a 'group' is an analysis of identical markers).

☒ All plots are contour plots with outliers or pseudocolor plots.

☒ A numerical value for number of cells or percentage (with statistics) is provided.

Methodology

Sample preparation	Single cell suspensions, generated as above, were incubated with Zombie Aqua™ Fixable Viability Kit (Biolegend, #423101, 1:1000) for 15 min at room temperature. Cells were washed with Cell Staining Buffer (Biolegend, #420201), then washed, centrifuged and 500xg for 5 min and blocked with Fc block CD16/32 (Biolegend, #101302, 1:100), for 5-minutes on ice and stained with PECAM1-BV605 (Biolegend, #102427, 1:1000, 100), CD45-FITC (Biolegend, #157607, 1:200), CD41-BV421 (Biolegend, #133911, 1:200), PDPN-eF660 (eBioscience, #50-5381-82, 1:100), LYVE1-PECy7 (eBioscience, #25-0443-82, 1:400) for 30-minutes on ice, then washed and resuspended in Cell Staining Buffer.
Instrument	Samples were either analysed immediately on a BD LSRFortressa X20 cytometer or stored in IC Fixation Buffer (eBioscience, #00-8222-49), washed and analysed the next day.
Software	Data were collected using FACSDiva (v8.0) software and analysed with FlowJo (v9) software.
Cell population abundance	FACS sorted cells were subjected to scRNA-seq or scMultiome-seq, demonstrating the degree of purity.
Gating strategy	Gating strategies are outlined in extended data figure 1. The initial SSC/FSC gating was broad and fluorescence minus one (FMO) controls were used for all antibodies/fluorophores.

☒ Tick this box to confirm that a figure exemplifying the gating strategy is provided in the Supplementary Information.
